# Mechanistic Insight into Bunyavirus-Induced Membrane Fusion from Structure-Function Analyses of the Hantavirus Envelope Glycoprotein Gc

**DOI:** 10.1371/journal.ppat.1005813

**Published:** 2016-10-26

**Authors:** Pablo Guardado-Calvo, Eduardo A. Bignon, Eva Stettner, Scott Allen Jeffers, Jimena Pérez-Vargas, Gerard Pehau-Arnaudet, M. Alejandra Tortorici, Jean-Luc Jestin, Patrick England, Nicole D. Tischler, Félix A. Rey

**Affiliations:** 1 Institut Pasteur, Unité de Virologie Structurale, Département de Virologie, Paris, France; 2 CNRS UMR3569 Virologie, Paris, France; 3 Fundación Ciencia & Vida, Molecular Virology Laboratory, Santiago, Chile; 4 Institut Pasteur, Ultrapole, Département de Biologie Cellulaire et Infection, Paris, France; 5 CNRS UMR 3528, Paris, France; 6 Institut Pasteur, Plateforme de Biophysique des Macromolécules et de leurs Interactions, Département de Biologie Structurale et Chimie, Paris, France; Purdue University, UNITED STATES

## Abstract

Hantaviruses are zoonotic viruses transmitted to humans by persistently infected rodents, giving rise to serious outbreaks of hemorrhagic fever with renal syndrome (HFRS) or of hantavirus pulmonary syndrome (HPS), depending on the virus, which are associated with high case fatality rates. There is only limited knowledge about the organization of the viral particles and in particular, about the hantavirus membrane fusion glycoprotein Gc, the function of which is essential for virus entry. We describe here the X-ray structures of Gc from Hantaan virus, the type species hantavirus and responsible for HFRS, both in its neutral pH, monomeric pre-fusion conformation, and in its acidic pH, trimeric post-fusion form. The structures confirm the prediction that Gc is a class II fusion protein, containing the characteristic β-sheet rich domains termed I, II and III as initially identified in the fusion proteins of arboviruses such as alpha- and flaviviruses. The structures also show a number of features of Gc that are distinct from arbovirus class II proteins. In particular, hantavirus Gc inserts residues from three different loops into the target membrane to drive fusion, as confirmed functionally by structure-guided mutagenesis on the HPS-inducing Andes virus, instead of having a single “fusion loop”. We further show that the membrane interacting region of Gc becomes structured only at acidic pH via a set of polar and electrostatic interactions. Furthermore, the structure reveals that hantavirus Gc has an additional N-terminal “tail” that is crucial in stabilizing the post-fusion trimer, accompanying the swapping of domain III in the quaternary arrangement of the trimer as compared to the standard class II fusion proteins. The mechanistic understandings derived from these data are likely to provide a unique handle for devising treatments against these human pathogens.

## Author Summary

Hantaviruses belong to the *Bunyaviridae* family of enveloped viruses. This family englobes in total five established genera: *Tospovirus* (infecting plants), and *Phlebovirus*, *Orthobunyavirus*, *Nairovirus* and *Hantavirus* infecting animals, some of which cause serious disease in humans. An important characteristic of the hantaviruses is that they are not transmitted to humans by arthropod vectors, as those from the other genera, but by direct exposure to excretions from infected small mammals. As all enveloped viruses, they require the activity of a membrane fusogenic protein, Gc, for entry into their target cells. Our structural analysis of the hantavirus fusion protein Gc led to the identification of a conserved pattern of cysteines involved in disulfide bonds stabilizing the Gc fold. This motif is matched exclusively by all of the available bunyavirus Gc sequences in the database, with the notable exception of phlebovirus Gc, which appears closer in structure to the fusion proteins of other families of arthropod-borne viruses, such as the flaviviruses and alphaviruses. This analysis further suggests mechanistic similarities with hantaviruses in the fusion mechanism of viruses in the remaining three most closely related bunyavirus genera, which we propose belong to a new separate sub-class of fusion proteins with a multipartite membrane targeting region.

## Introduction

Hantaviruses are a small group of zoonotic viruses of rodents, bats and insectivores such as moles and shrews [1]. They are often transmitted to humans by persistently infected rodents, causing serious outbreaks of pulmonary syndrome or of hemorrhagic disease with renal syndrome [2, 3]. The case fatality rates can reach 50%, for instance in the case of the “Sin Nombre” hantavirus outbreak in the 1990s in the four-corners area in the US [4]. The name hantavirus derives from the prototype virus, Hantaan virus, which was discovered in the early 1950s during the Korean war, when troops stationed by the Hantaan river developed hemorrhagic manifestations [5]. Outbreaks of hantavirus disease of varying severity have occurred periodically in the last decades throughout the Americas [6, 7] and in Europe and Asia [8, 9]. It is therefore important to understand the structural organization of hantavirus particles as a step forward in attempts to devise curative or preventative strategies.

Hantaviruses constitute one of five genera forming the *Bunyaviridae* family of enveloped RNA viruses, which have a genome composed of three segments of single-stranded RNA of negative polarity [10]. The bunyavirus proteins involved in genome replication–the large (L) polymerase and the nucleocapsid (N) protein, encoded in the large and small genome segment, respectively–are very similar to their counterparts in other families of segmented negative-strand (sns)RNA viruses, such as the *Arenaviridae* or the *Orthomyxoviruses* [11]. In contrast, the envelope glycoproteins, which derive from a polyprotein precursor encoded in the medium (M) size genomic segment, are totally unrelated. Whereas the other snsRNA virus families display class I membrane fusion proteins characterized by a central alpha-helical coiled-coil in their post-fusion form, the bunyavirus envelope proteins have properties of class II enveloped viruses [12]; i.e., they are β-sheet-rich proteins (as predicted from their amino acid sequences) such as those found in the icosahedrally symmetric flaviviruses and alphaviruses. The latter, like viruses in the Bunyaviridae family other than the hantaviruses, are transmitted to vertebrates (or to plants in the case of tospoviruses) by insect or tick vectors, and are accordingly termed arthropod-borne viruses or “arboviruses”.

In class II viruses, the membrane fusion protein is the second in a tandem of proteins–Gn and Gc in the bunyaviruses—encoded sequentially within a single precursor polyprotein. They heterodimerize in the ER and are then transported to the site of budding, which is the Golgi apparatus for a number of bunyaviruses [13], although some hantaviruses were reported to bud at the plasma membrane [14, 15]. The bunyavirus Gc glycoproteins were predicted to adopt a “class II” fold using proteomic computational analyses [16], and Gc from Andes virus (a hantavirus endemic in South America) was modeled using the flavivirus E protein as template [17], thereby predicting the location of the “fusion loop”, which was later functionally confirmed as important for fusion by site directed mutagenesis [18]. Similarly, for La Crosse virus in the *Orthobunyavirus* genus, mutagenesis of the predicted fusion loop region—deduced by comparison to the alphavirus fusion glycoprotein E1—confirmed the importance of this region for entry [19]. More recently, the determination of the crystal structure of Gc of the Rift Valley Fever virus [20], a bunyavirus in the *Phlebovirus* genus, demonstrated that it indeed has the typical fold of class II membrane fusion proteins, providing a conclusive proof. These observations are compatible with the icosahedral organization of the phleboviral particles observed by electron microscopy [21, 22]. Extrapolation to the other genera should be done with caution, however, since the sequence similarity is very low and not easily detectable by current methods. In the case of the *Flaviviridae* family, for instance, where viruses outside the *Flavivirus* genus were also expected to have class II fusion proteins, the prediction was proven wrong when the corresponding crystal structures were determined, i.e. the E2 protein from the pestiviruses [23, 24] and from the hepatitis C virus [25, 26].

Structural studies on members of the *Bunyaviridae* family are relatively limited, providing the low-resolution overall arrangement of spikes at the virus surface, but not the organization of the individual proteins. Differently to the icosahedral organization observed in virions of the *Phlebovirus* [21, 22] and *Orthobunyavirus* [27] genera, hantavirus particles were shown to be pleomorphic, with some virions showing a roughly spherical and others an elongated aspect [28]. Cryo-electron tomography and sub-tomogram averaging revealed that the Gn/Gc spikes are arranged with apparent 4-fold symmetry [29, 30] on a curved square lattice, incompatible with icosahedral symmetry. The resulting array of hantavirus spikes does not cover the whole surface of the observed particles. The organization of the Gn/Gc subunits within the spikes was interpreted as a (GnGc)_4_ hetero-octamer. A recent study described the crystal structure of the Gn ectodomain of Puumala hantavirus, which was docked on the spike using the 3D reconstruction of Tula hantavirus extended to 16Å resolution, placing Gn at an exposed site on the spike, distal to the viral membrane [31].

Here, we report the crystal structure of the ectodomain of Gc from Hantaan virus in a monomeric pre-fusion conformation and in its trimeric post-fusion form, validating its prediction as a class II fusion protein, albeit presenting a number of hantavirus-specific features. Combined with structure-guided functional studies on the related Andes virus, we show that hantavirus Gc has a multi-partite membrane interaction surface, with residues outside the *cd* loop (which is the fusion loop in standard class II proteins) also being important for membrane insertion and fusion. We further show that Gc requires the formation of a carboxylate-carboxylic acid hydrogen bond, which can form only at acidic pH, for structuring the membrane interacting region. In addition, we show that hantavirus Gc has an N-terminal segment (the “N tail”) which is absent in other class II proteins and which is functionally involved in trimerization to form the stable post-fusion form. Further analysis also identifies a conserved “cysteine” signature in the amino acid sequence of Gc giving rise to a disulfide bonding pattern conserved in the *Nairovirus*, *Orthobunyavirus* and *Tospovirus* genera, but notably different from the phlebovirus Gc.

## Results

### The hantavirus Gc ectodomain is monomeric in solution

We expressed the recombinant ectodomain of Gc (rGc) from Hantaan virus strain 76–118 (UniProtKB/SwissProt accession: P08668.1) in *Drosophila* S2 cells as described in Materials and Methods. It behaved in solution as a monomer, both at neutral and acidic pH, as assessed by size exclusion chromatography (SEC) and multi-angle static light scattering (MALS) (S1A Fig). Electron micrographs of negatively stained samples of purified rGc showed a thin rod-like molecule of ~140Å in length (see below). Despite multiple trials, rGc failed to crystallize on its own, and we therefore screened a human single-chain variable domain (scFv) antibody fragment library, which in principle was hantavirus-naïve [32], to identify potential binders that could act as crystallization chaperones. We thus identified scFv A5, which is very close to its germ line (Table 1) and which interacted with rGc as monitored by ELISA (see Methods). Further analysis by size-exclusion chromatography (SEC) together with multi-angle light scattering (MALS), showed that scFv A5 forms a 1:1 complex with rGc (S1B Fig).

**Table 1:** Germ line analysis of scFv A5.

	V-H/V-L allele	V-H /V-L	V-H/V-L aa	J-H allele	D-H allele	CDR length	BASA (%)	
		diverg^||^	ch/tot^¶^			**[1:2:3]**	**CDR[1:2:3]**	**[CDR:FWR]**
**Heavy chain**	IGHV4-4*02	1.38% (4/288)	3/98	IGHJ1*01^†^	IGHD2-2*01	[9:7:6]^§^	[14:20:6]^§^	[40:1]^§^
**Light chain**	IGLV3-19*01	0.00% (0/279)	0/96	IGLJ2*01^†^	-	[6:3:11]^§^	[33:4:14]^§^	[51:8]^§^

V-H, J-H, D-H. V-L, J-L represent the putative human genes and alleles predicted by IMGT analysis [33].

^†^Additional possibilities were also predicted by IMGT

^||^Nucleotide (nt) divergence (diverg.). The total length for all V-H and V-L alleles is 288 nt for heavy chain and 279 nt for light chain.

^¶^Number of amino acid (aa) changes out of total V-H/V-L aa length (ch/tot).

BASA: (buried accessible surface areas) calculation for CDR and FRW regions shown as percentage of the total BASA of ScFv within the complex and per individual H-CDRs [1:2:3] and L-CDRs[1:2:3] or as a sum for light/heavy chain CDRs and FWRs [CDR:FWR].

^§^IMGT definition of CDR and FRW regions.

**Fig 1:**
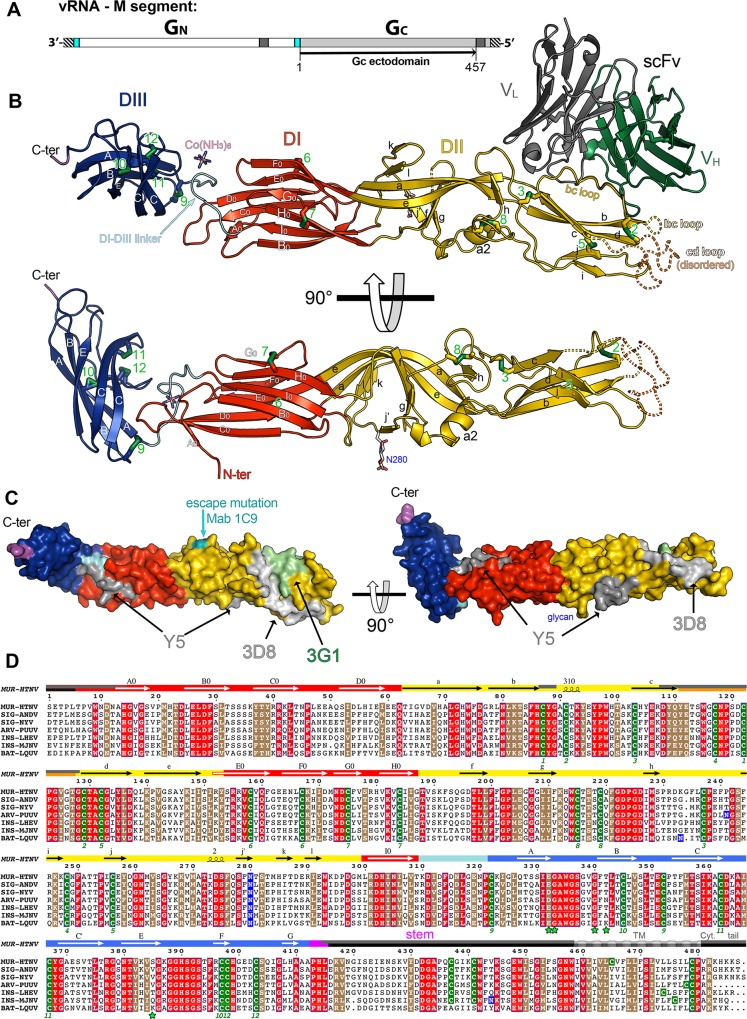
Structure of Hantaan virus Gc. A) Organization of the hantavirus M genomic RNA segment. The black striped regions represent the non-coding 3’ and 5’ RNA ends, with the single open-reading frame in between. Regions corresponding to the signal sequences for Gn and Gc are in cyan, and other trans-membrane regions are in dark grey. The black line below indicates the region spanned by the recombinant Gc ectodomain (rGc). B) The rGc / scFv A5 complex. Gc is colored according to domains with domains I, II, and III in red, yellow, and blue, respectively, with secondary structure elements labeled. The linker between domains I and III is shown in cyan, and the C-terminal end in magenta (beginning of the “stem” region). The disulfide bonds are displayed as sticks with sulfur atoms in green, and numbered in green. The scFvA5 is shown with the heavy chain dark green and the light chain in grey. The lower panel shows an orthogonal view of Gc, with the scFvA5 removed for clarity. The single N-linked glycan Asn280 is labeled. Dotted lines indicate the disordered region, corresponding to the *cd* loop (orange) and the distant end of the *bc* loop (yellow). C) The Gc surface colored according to domains, with the epitopes of—and sites of escape mutation to—neutralizing antibodies mapped in grey or green in different shades. The protein is shown in the same two orthogonal views of B. D) Multiple sequence alignment of Gc from seven representative hantaviruses. Strictly conserved and highly similar residues are highlighted in red or brown background, respectively. All the cysteines are shown in a green background, and asparagine residues within N-linked glycosylation motifs in a blue background. The corresponding host is noted as MUR (*Murinae*), SIG (*Sigmodontinae*), ARV (*Arvicolinae*) corresponding to rodent subfamilies and as INS (Insectivores), and BAT (*Chiroptera*). The secondary structure elements are displayed above the sequences, on a background colored according to the tertiary structure, as in panel B. The missing regions are in grey or black (within the colored tertiary structure bar, a top grey half bar denotes residues disordered in the neutral pH structure, and a bottom black half bar those not visible in the post-fusion structure). A hashed grey bar denotes the TM segment. Disulfide bonds are numbered in green below the sequences. The domain III residues involved in inter-subunit interactions with the Asn280 glycan in the post-fusion trimer are marked with a green star under the alignment.

We obtained crystals diffracting to 3Å resolution of the A5/rGc complex at pH 7.5, after limited proteolysis of the complex with trypsin. This treatment removed a C-terminal segment of rGc and the purification tags of both, A5 and rGc. We determined the X-ray structure by a combination of molecular replacement with the variable domains of an antibody and the anomalous scattering from a samarium derivative (see Materials and Methods). The final electron density map was clear for amino acids (aa) 16–414 of rGc (out of residues 1–457 in the intact Gc ectodomain, Fig 1A), with internal breaks at loops 84–91 and 108–132 (Fig 1B). These disordered segments map to the tip of domain II, at the membrane interacting region, as discussed below. All A5 residues were clearly resolved in density, except for the linker connecting light and heavy chains in the scFv construct. An atomic model of the complex was built into the electron density with the program Coot and refined with Phenix.refine to an R factor of 22% and free R factor of 27% (S1 Table). The scFv makes an important contribution to the packing contacts in the crystal. The antibody buries about 1,000 Å^2^ of the accessible surface of rGc, with 60% of the contacts made by the light chain (which is un-mutated with respect to its germ line, see Table 1). In spite of the relatively large buried antibody/antigen surface, affinity measurements by surface plasmon resonance (SPR) indicated an A5:rGc binding constant in the micromolar range, in line with the absence of affinity maturation of the human scFv library (S1C and S1D Fig).

### Hantavirus Gc has a typical class II fold

The structure shows that Gc displays a typical class II fusion protein fold (Fig 1), first observed in the ectodomain of the flavivirus E [34] and alphavirus E1 [35] fusion proteins, and more recently in the rubella virus E1 glycoprotein [36] and in phlebovirus Gc [20]–the only other genus of the *Bunyaviridae* family for which a Gc structure is available. The class II fold features a central β-sandwich domain (termed domain I) made of eight β-strands labeled B_0_ through I_0_, connected sequentially with up-and-down topology and arranged in two antiparallel β-sheets, the “inner” B_0_I_0_H_0_G_0_ and the “outer” C_0_D_0_E_0_F_0_ sheets apposed against each other (the names of the β-sheets refer to their orientation in the post-fusion trimer). Like flavivirus E and phlebovirus Gc, hantavirus Gc has an additional short N-terminal β-strand, A_0_, which starts at residue 18 and runs parallel to strand C_0_ at the edge of the outer sheet. The N-terminal segment, upstream of strand A_0_ and which contains a number of residues strictly conserved across the *Hantavirus* genus (Fig 1D), is disordered in the crystals.

The segments connecting β-strands D_0_ to E_0_ in the outer sheet and H_0_ to I_0_ in the inner sheet are very long and elaborated. They make up domain II (yellow in the Figures), which is composed of 13 β-strands (labeled *a* through *l*) and a couple of short helices (η1 and α2, Fig 1). Strand *j’*, unique to hantavirus Gc and inserted between helix α2 and strand *k* (Fig 1D) carries the single Gc N-linked glycosylation site at Asn280 (Fig 1B), which is strictly conserved across hantaviruses (Fig 1D). Domain II has an elongated shape with two subdomains, a central, domain I-proximal open β-barrel made of β-strands *klaefgj’*, and a distal “tip”—a β-sandwich between the *bdc* β-sheet and the *ij* β-hairpin, which projects the *cd* loop (which is the fusion loop in the standard arbovirus class II proteins) at its distal end. This region corresponds to the disordered Gc tip in the crystal structure, stabilized in part by the scFv A5 (Fig 1B). Finally, after strand I_0_, domain I connects via a 12 residue linker (cyan in Fig 1) to domain III (blue), which has an immunoglobulin superfamily C2 subtype fold [37] composed of β-strands A through G (Fig 1B and 1D).

Hantavirus Gc has a total of 26 cysteine residues in the ectodomain, 24 of which are present in the crystallized fragment, making 12 disulfide bonds. The surface area buried at the interface between domains I and III is very small, and is stabilized in the crystals by a cobalt-hexamine ion (Fig 1B), which was identified in crystal optimization screens to improve the diffraction of the crystals.

### Epitopes on Gc

Although Gn was shown to be displayed prominently on the hantavirus spikes, and some of the epitopes of neutralizing antibodies were mapped there [31], a number of hantavirus neutralizing antibodies target Gc as well. The only neutralization escape mutant mapping to Gc reported in the literature corresponds to an S287F change in Puumala hantavirus Gc and confers escape from neutralizing Mab 1C9 [38], which maps to the *k* strand on one side of domain II (Fig 1C). There are also data on Gc residues that affect binding of neutralizing antibodies for various other hantaviruses. Although often such epitopes are conformational and cannot be mimicked by peptides, the epitope of the neutralizing murine Mabs 3G1 and 3D8 [39] against Hantaan virus have been respectively mapped by peptide scanning to residues 96–105 [40] and 242–248 [41]. The 3G1 epitope thus maps to the *bc* loop and largely overlaps with that of scFv A5, whereas the 3D8 epitope maps to the *i* strand, at the opposite side (Fig 1C). The epitope of a human antibody against Hantaan virus, Y5, was also identified by peptide scanning, and mapped to two discontinuous segments of the Gc polypeptide, 268–276 and 307–315 [42], which correspond, respectively, to the region around helix α2 in domain II and to the end of strand I_0_ and the linker between domains I and III (see Fig 1C and 1D). These two segments are far apart on the Gc monomer, but may be located more closely in a multimeric arrangement of Gc on the hantavirus spike. Although the docking of Gn appeared to be clear in the reported 16Å resolution electron density map of Tula hantavirus [31] and allowed to understand how the Gn epitopes are exposed on the spike, docking rGc is less clear in the same map and will need to await higher resolution data and/or a crystal structure of a Gn/Gc heterodimer to unambiguously assess where the Gc epitopes lie on the spike. In particular, as the epitopes map toward the Gc tip, which is partially disordered in the crystals, the conformation of this flexible region may be affected by Gn-Gc interactions on the spike.

### Interaction of rGc with lipids

**Fig 2:**
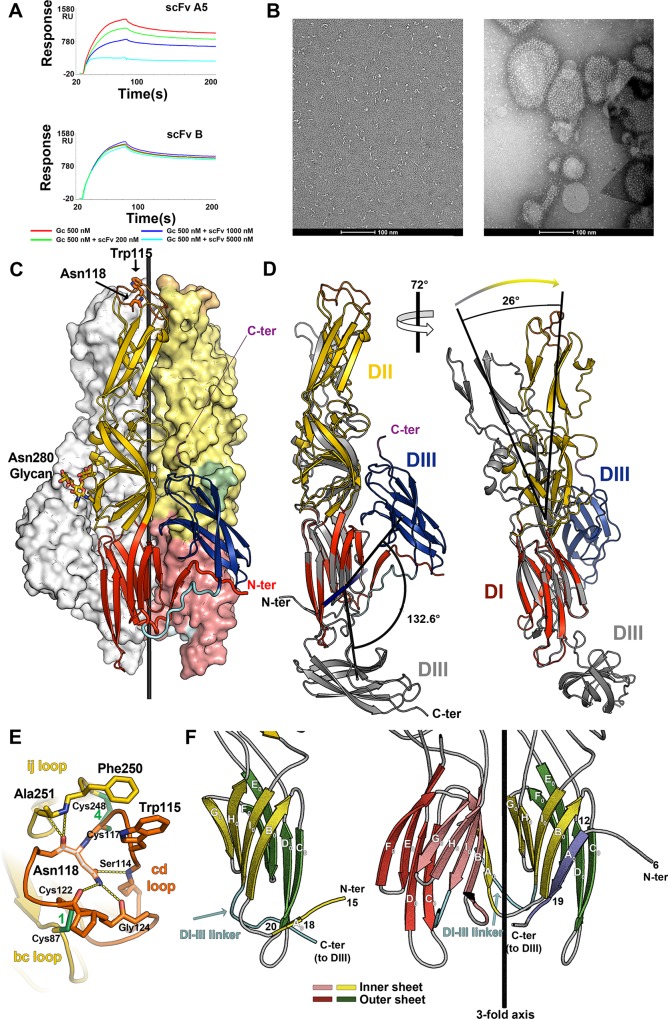
Gc interactions with lipids and post-fusion structure. A) scFv A5 specifically inhibits liposome binding by rGc as measured by SPR. The liposomes were immobilized on an SPR chip (see Methods) and Gc alone or together with scFv A5 at increased concentrations was injected to measure binding (top panel). The same experiment with an unrelated, non-binding scFv is provided as control in the lower panel. B) Left panel: Electron micrographs of negative stained samples of rGc show long thin rods with dimensions comparable to the neutral pH structure shown in Fig 1B and 1C. Right Panel: Incubation of rGc with liposomes (see Materials and Methods for lipid composition) at low pH results in rGc insertion into the membranes, with a concomitant change in aspect to resemble class II viral fusion proteins inserted into liposomes in their post-fusion, trimeric conformation (as shown previously for alphaviruses and flaviviruses). C) The structure of the Gc trimer crystallized at pH 6.5 (W115H mutant) displays the typical class II post-fusion conformation. The protomer in the foreground is shown in ribbons colored by domains, with the single glycosylation site at Asn280 in sticks, as well as key residues at the tip of domain II. The two protomers in the background are shown as surfaces, one colored in grey and the other by domains, with the glycan surface in green. The structure determination used a W115H mutant, but the representation shows the location of the mutated tryptophan (labeled). The trimer axis is drawn in black. D) Conformational transition of rGc. The neutral pH monomeric form colored gray and the low pH trimer subunit colored according to domains were superposed on domain I and are shown in two views to highlight the overall reorganization of the molecule. The left panel shows the axis (nearly normal to the plane of the Figure) about which domain III pivots by more than 120 degrees. The right panel shows the 26 degrees hinge of domain II about the domain I/II junction. E) Close up view (from the top) of the tip of domain II showing the structuring role of Asn118. The polypeptide chain is shown in yellow except for the *cd* loop (orange), with non-carbon atoms in different color (nitrogen, oxygen and sulfur in blue, red and green, respectively). Asn118 is highlighted in white sticks with an orange outline. Its side chain carboxamide group donates two hydrogen bonds (yellow dots) to the main chain carbonyls of Cys122 and Gly124, and accepts a hydrogen bond from the main chain amide of Ser114. In addition, the main chain carbonyl of Asn118 accepts two hydrogen bonds from the *ij* loop, from the main chain amide groups of Ala251 and Thr252. Note that Asn118 is framed by disulfides 4 (immediately upstream) and 1 (downstream), which connect the *cd* loop to the *ij* and *bc* loops, respectively. Trp115 and Phe250, discussed in the paper, are displayed in sticks, as a guide. F) Cartoon to show the swapping of β-strand A_0_ during trimerization. The left panel shows domain I in the pre-fusion conformation, with inner and outer β-sheets in yellow and green, respectively. The short A_0_ strand runs antiparallel to C_0_ at one edge of the outer β-sheet (indicated in yellow to highlight that it becomes part of the inner sheet in the post-fusion form). In the trimer, the A_0_ strand swaps to the adjacent domain I (depicted with inner and outer β sheets in pink and red, respectively), to run antiparallel to the B_0_ strand in the inner β-sheet. For clarity, the front subunit in the trimer (which would have inner and outer sheets in pale and dark blue) was removed, leaving only its A_0_ strand, which inserts into the β-sandwich of the yellow/green domain (on which the monomer was superposed for this view). The “pink” A_0_ strand inserts into the blue subunit (not displayed for clarity). Downstream strand I_0_, the domain I-III linker runs alongside the inserted A_0_ strand in the trimer, swapping also domain III, which is further downstream (see panel C). The limits of strand A_0_ are labeled, to show what residues rearrange (see also the secondary structure diagram in Fig 1D).

We investigated the behavior of rGc in interaction with lipids, and found that it binds liposomes at acidic pH as detected by flotation on sucrose gradients and by SPR on a matrix with immobilized liposomes (see Materials & Methods) (S2A Fig). The SPR measurements also detected binding to liposomes at pH 7.4 (S2B Fig), although to a lower extent than at pH 5.5. We found that the interaction required the presence of cholesterol in the liposomes (Fig 2A), in agreement with recent studies [43]. Addition of the scFv A5 interfered with lipid binding (Fig 2A), in line with its epitope lying close to the membrane interacting region of Gc (Fig 1). Because the A5 epitope overlaps with that of the neutralizing Mab 3G1, our data suggest that the neutralization mechanism of antibodies targeting this region involves blocking membrane insertion of Gc.

Electron microscopy showed that the Gc proteoliposomes display radial projections with a shorter and thicker aspect than the observed overall shape of monomeric rGc in solution (Fig 2B). The rGc projections on the liposomes are very similar in size and shape to the trimeric projections made by class II viral fusion proteins in their post-fusion conformation on liposomes [36, 44, 45]. This similarity suggested that rGc had adopted its predicted post-fusion conformation, as recently evidenced for Andes virus Gc by sucrose sedimentation in an *in vitro* system [46]. Because attempts to crystallize the membrane inserted form of rGc failed, we introduced a mutation in the predicted fusion loop, which caused rosette formation upon concentration after detergent solubilization (in line with the model for insertion of trimeric post-fusion class II proteins into membranes, reviewed in [47, 48]). The fusion loop mutation was inspired by a recent report showing that the trimeric post-fusion form of the flavivirus class II fusion protein E could be crystallized in its post-fusion, trimeric form in the absence of detergent by replacing Trp 101 by histidine, as this residue is prominently exposed at the membrane facing-end of the post-fusion trimer [49]. We accordingly substituted Trp115, predicted to be at the tip of the *cd* loop in hantavirus Gc [18] by histidine. The rGc W115H mutant indeed crystallized under mildly acidic conditions (pH 6.5) in the rhombohedral space group R3, and the crystals diffracted to 1.6Å resolution.

### The post-fusion form of Gc

We determined the crystal structure of acid-pH rGc by molecular replacement using the individual domains of Gc, and refined the atomic model to 1.6 Å resolution to an R factor of 14% (free R factor 17%) (S1 Table). A single Gc protomer (or trimer subunit) is present in the asymmetric unit of the crystals, packing about a crystallographic 3-fold axis and adopting the characteristic class II post-fusion form (Fig 2C). As in the other class II proteins [36, 50–53], the post-fusion form shows a drastic re-orientation of domain III such that it packs laterally against the domain I/II junction of both, the same and the adjacent subunits in the trimer (Fig 2C). This relocation of domain III is in line with recent data showing that exogenous domain III can block the fusion process [54] by binding to the domain I/II inner trimer core and interfering with the necessary translocation of domain III to reach the post-fusion hairpin conformation, as had been shown earlier for alpha- and flaviviruses [55]. The Gc structures show that during the pre- to post-fusion transition, domain II hinges by 26 degrees about the domain I/II junction, thereby bringing the domain II tips of the three protomers into contact at the trimer tip (Fig 2D). The A5 epitope remains accessible on the trimer, and modeling shows that three scFvs can bind simultaneously to one trimer (S2C Fig). The observed inhibition of trimer insertion is likely due to the scFv protruding further at the tip of the trimer than the fusion loop itself, helping the complex to remain in solution.

The buried area per Gc subunit in the trimer is 2290 Å^2^, and the residues at the interface are mostly hydrophilic and conserved (S3 Fig). In contrast to the neutral pH form, the tip of domain II, (the *cd* loop but also the neighboring parts of the *bc* and *ij* loops) displayed clear electron density and allowed tracing the polypeptide chain with no breaks. As expected, the side chain of Trp115 (which is His115 in the mutant used for the crystals) is exposed at the very tip of domain II, where it would be expected to insert into membranes. In a previous study, Trp115 was indeed shown to be essential for fusion activity of Andes hantavirus [18]. That study also showed that Asn118 in the *cd* loop was essential for membrane fusion. The low pH structure now shows that the Asn118 side chain makes a crucial set of hydrogen bond interactions with the peptide backbone of the *cd* loop and its main chain with the *ij* loop (Fig 2E). This key structuring-role of the domain II tip explains the strict conservation of Asn118 across hantaviruses (Fig 1D) and its functional importance for fusion.

### A carboxylate-carboxylic acid hydrogen bond is critical to structure the tip of domain II

**Fig 3:**
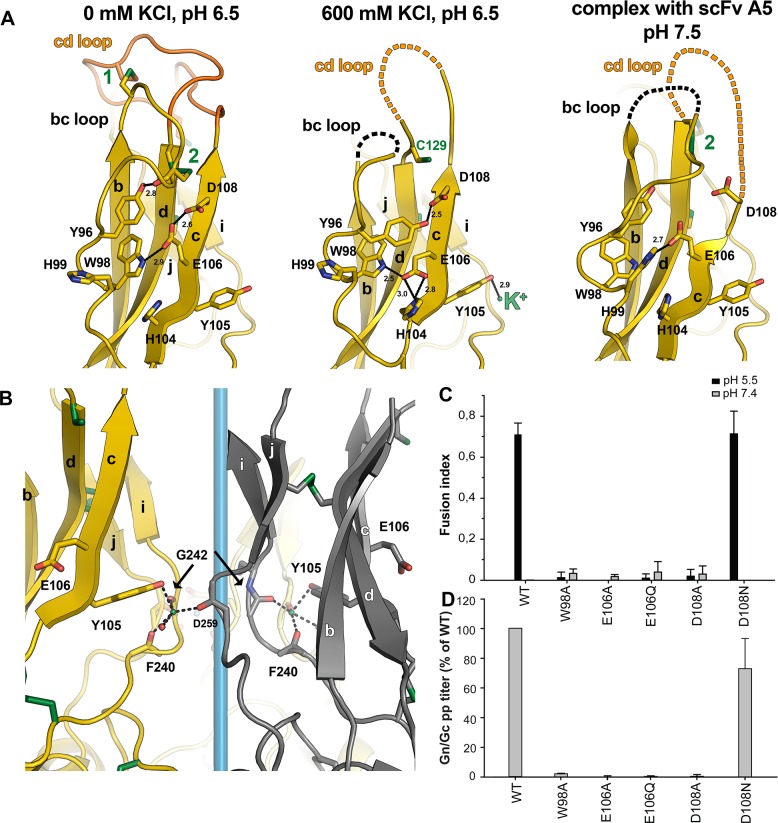
A carboxylate-carboxylic acid hydrogen bond structures the domain II tip. A) Left panel: the Glu106 side chain is at the center of a string of hydrogen bonds linking the side chains of Asp108 and Trp98 through that of Glu106. The overall network also involves the phenol group of Tyr96 making a hydrogen bond with the main chain carbonyl of Cys91 in the *bc* loop (involved in disulfide bond 2, connecting the *bc* loop to the *d* strand) and via a water molecule to the imidazole ring of His86 (located at the end of the *b* strand, not displayed for clarity). See the corresponding electron density in the matching S1 Movie. Middle panel: the shift in position of Tyr105 to chelate the K^+^ ion affects Glu106, such that its pK drops and becomes deprotonated. It now accepts a bifurcated hydrogen bond from the imidazole ring of His104, while the phenol group of Tyr96 now donates a hydrogen bond to Asp108, releasing a set of interactions that lead to de-structuring of the *cd* loop and the N-terminal part of the *bc* loop (dotted lines). In particular, the side chain of Cys91 becomes disordered, and only the side chain of its partner in disulfide bond 2 remains partially visible (Cys129). The corresponding electron density in shown in the S2 Movie. Right panel: the tip of domain II at neutral pH from the structure of the complex with scFv A5 (which was removed, for clarity). Although the resolution of this structure is lower (3 Å instead of 1.6 Å and 1.4 Å) and the hydrogen bond interactions are less well defined than in the structures of the Gc trimer, it is clear that at neutral pH Glu106 accepts a hydrogen bond from His99. The corresponding electron density is shown in the S3 Movie. Asp108 is the last residue with a visible side chain, and is too far to interact with E106. At this pH, such an interaction is not expected, since both side chains will be negatively charged and repel each other. B) Close view of the K^+^ binding site, near the 3-fold axis on the Gc trimer when crystallized in the presence of KCl at concentrations above 200 mM. The reference protomer is colored yellow and an adjacent one dark grey. The third protomer is not displayed, for clarity, and the 3-fold axis is represented in light blue. β-strands are labeled. The K^+^ ion is shown as a small green sphere, and the residues involved in its coordination are depicted in sticks, as well as Glu106, which forms the carboxylate-carboxylic acid hydrogen bond at low pH in the absence of KCl. Except for Tyr105, the K^+^ coordinating atoms are all from the main chain. The various crystal structures at different KCl concentrations are summarized in the S4 Fig and in the S4 Movie. C) and D) Andes virus Gc mutants in the residues involved in the side chain Asp108-Glu106-Trp98 hydrogen bond network are impaired in membrane fusion activity. The expression level and plasma membrane localization of these mutants (which concern residues strictly conserved in the hantavirus genus, Fig 1D) is provided in the S5 Fig, which shows that they reach the cell surface in similar amounts as wild type. C) Syncytia formation of cells expressing Gn and wild type or mutant Gc when exposed at the indicated pH. Fluorescence microscopy employing three different stains was used for the quantitation of cells and nuclei and syncytia formation results from at least n = 2 experiments were averaged (see the S6 Fig). D) Entry of Vero E6 by SIV particles pseudotyped with Andes virus spikes with wild type Gn and either Gc wild type or the indicated mutant (Gn/Gc pp). Reporter gene (GFP) expressing cells were quantified by cell cytometry (S7 Fig).

In addition to the role of Asn118 in structuring the fusion loop, we observed that the disorder at the tip of domain II in the monomeric, pre-fusion form of Gc begins at residue Asp108 in β-strand *c*, within a strictly conserved 106-EXD-108 amino acid motif (where X is any amino acid) (Fig 1D). In the post-fusion structure, the side-chain of Glu106 is connected via hydrogen-bonds to the indole ring of the strictly conserved Trp98 and to the Asp108 side chain (Fig 3A, left panel), making a relatively short (2.6 Å distance) carboxylate—carboxylic acid hydrogen bond. The pK of the amino acids in the latter interaction is therefore shifted in the structure, which was obtained at pH 6.5 (see S1 Table) (the normal pK of glutamic and aspartic acid is 4.2 and 3.8, respectively), such that a proton is present in between. Of note, in crystals of rGc also at pH 6.5 obtained in the presence of KCl at concentrations above 200 mM, Gc was in its post-fusion form but the tip was disordered (S4 Fig). The crystal-packing environment was not responsible for the observed disorder, as the crystals were isomorphous, having the same symmetry and the same cell parameters, and diffracting to high resolution (around 1.4 Å).

Inspection of the structure further showed that a K^+^ ion from the crystallization conditions becomes trapped near the central 3-fold axis of the rGc trimer, coordinated by the side chain hydroxyl group of Tyr105 together with the main-chain carbonyl oxygens of Phe240, Gly242 of the same subunit and of Asp259 from a neighboring protomer, as well as an immobilized water molecule (Fig 3B). Tyr105 directly precedes the di-carboxylate 106-EXD-108 motif, and as it adjusts its orientation to chelate the K^+^ ion, it alters the main chain such that Glu106 is pulled away from the interaction with Asp108, becoming de-protonated and now accepting hydrogen bonds from the imidazole ring of His104, while still maintaining the interaction with the indole ring of Trp98 (Fig 3A, middle panel). These results therefore indicate that formation of the carboxylate-carboxylic acid hydrogen bond is essential to the organization of the tip of domain II, and that the reason why this region is disordered in the neutral pH structure is that this interaction cannot form (Fig 3A, right panel). This disordered region in Gc at neutral pH is in line with the altered mobility of Gc in SEC at the two pH values measured, with the monomer at neutral pH displaying a larger Stokes radius for the same molecular mass (S1A Fig).

We tested the functional relevance of these interactions in the Andes hantavirus system, for which it was shown that expression of the M genomic segment (i.e., coding for wild type Gn and Gc proteins) in cells induces syncytium formation when treated at low pH. We thus introduced each of the following Gc substitutions: E106A, E106Q, D108A, D108N and W98A into Andes virus Gc using this plasmid, and compared syncytium formation by the mutants and by the wild type protein. Although the level of Gn and Gc that reached the cell surface was similar to wild type (S5 Fig), there was no syncytium induced by the mutants except for D108N (Fig 3C), which can still make a hydrogen bond with the Glu106 side chain (Fig 3A, left panel). The reverse situation, in the E106Q mutant, is not viable, indicating that the Glu106 side chain is essential in this process, perhaps because of its dual interaction with Asp108 and Trp98 (Fig 3A, left panel). We confirmed these results by introducing the same mutations into a system of SIV particles pseudotyped with the Andes virus glycoproteins, which allows visualization of entry by expression of a fluorescent reporter gene [56]. Again, only the D108N mutant was as efficient as wild type for entry (Fig 3D), whereas none of the other mutants was, in spite of being present in similar amounts as wild type Gn and Gc on these particles (S5 Fig). The requirement for a glutamic acid to be protonated in order to organize the structure of the domain II tip only upon acidification is unique to hantavirus Gc, as it has not been described so far for any other membrane fusion protein.

### The Gc N tail and its interactions with domain III

**Fig 4:**
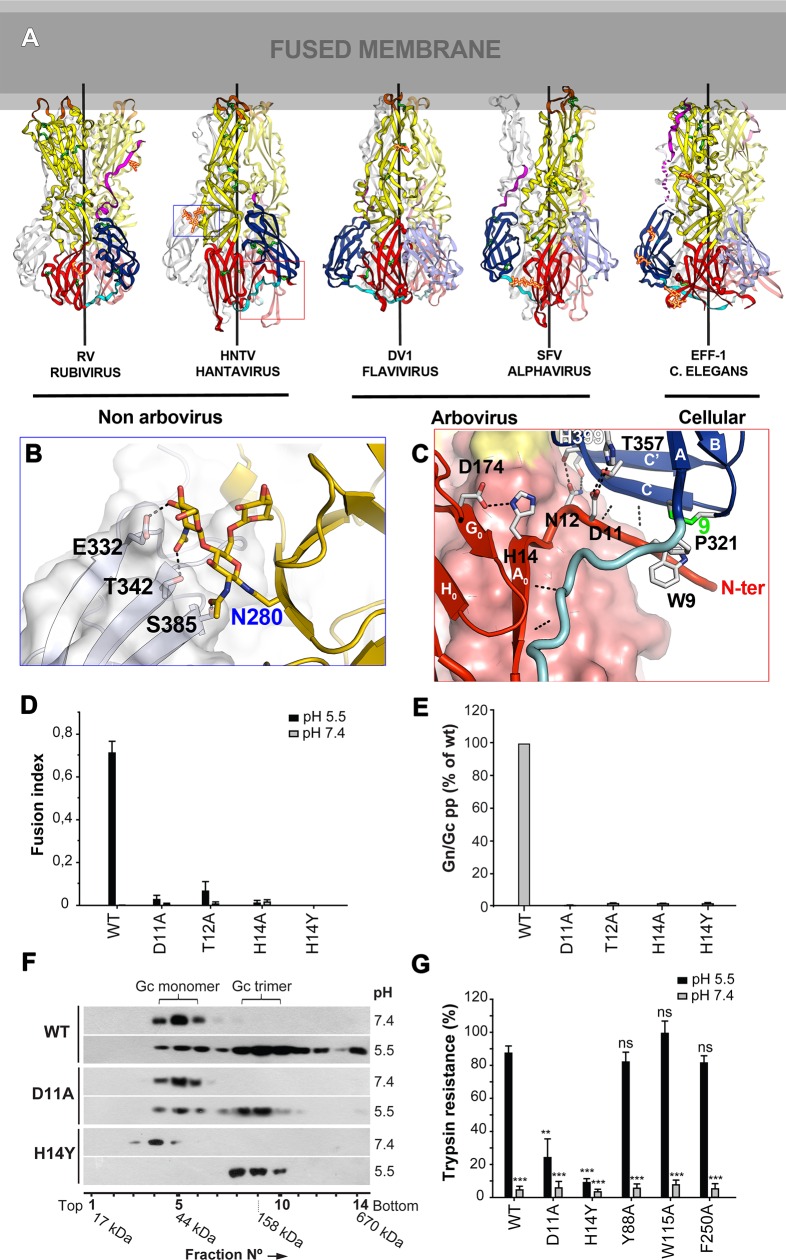
Domain III is “swapped” in the post-fusion Gc trimer with respect to the standard arbovirus class II fusion proteins. A) Comparison of the post-fusion Gc trimer with the other class II fusion proteins of known structure. A cartoon representation of Hantaan virus Gc next to the rubella virus E1 (labeled RV; PDB entry 4B3V [36]) to its left, and Semliki Forest Virus E1 (alphavirus, labeled SFV; PDB entry 1RER [51]), the Dengue virus serotype 1 E protein (flavivirus, labeled DV1; 4GSX [49]), and the *C*. *elegans* cellular fusogen EFF-1 (4OJC [59]). A « fused membrane » is diagramed above, with aliphatic and hydrophilic layers in dark and light gray, respectively. In each trimer, a “reference” subunit is shown in bright standard class II colors (with the stem region in dark pink), the anticlockwise subunit when looking from the membrane in pale colors, and the third in light grey. Disulfide bonds are green, and glycan chains are shown in gold sticks with a red outline. The trimer axes are drawn in each case. Note that the non-arthropod-borne rubiviruses and hantaviruses have domain III swapped with respect to the others (i.e, the dark blue domain III is to the right and not to the left, as in the others). Note also two features specific of hantavirus Gc: the N tail (boxed in red) interacting with the domain I-III linker and with domain III, and the Asn280 glycan interacting with a neighboring protomer (boxed in blue). B) Close up view showing the inter-subunit contacts of the sugar residues within the trimer. The domain III amino acids interacting with the glycan are also marked with green stars in the alignment of Fig 1D. C) Close up view of the polar contacts of conserved residues from the N tail. Hydrogen bonds are indicated as dotted lines, including those between main chain atoms, in which case the interacting atoms are not drawn but only the ribbon, for clarity. D-E) Mutation of conserved N tail residues render Gc non-functional for fusion. D) Fusion activity of cells expressing Gn and N tail mutants of Andes virus Gc at neutral or acid pH. Syncytia formation was quantified by counting cells and nuclei using three-color fluorescence microscopy (S6 Fig) and results from at least n = 2 experiments were averaged. E) Entry of Vero E6 by SIV particles pseudotyped with Andes virus spikes (Gn/Gc pp) with wild type Gn and Gc wild type or the indicated N tail mutant. Reporter gene (GFP) expressing cells were quantified by cell cytometry (S7 Fig). F-G) trimer formation and stability of selected N tail mutants of Andes virus Gc. F) Acid-induced trimer formation of wild type or mutant Gc. Sucrose sedimentation of Andes virus VLPs after treatment at the indicated pH and subsequent extraction by Triton X-100. The presence of Gc in different fraction was detected by western blot analysis and the molecular mass of each fraction determined by a molecular marker. G) Trimer stability of wild type and mutant Andes virus Gc assayed by trypsin. VLPs including Gn and wild type or mutant Gc were treated at the indicated pH for 30 min, back-neutralized, and incubated with trypsin for 30 min. Gc resistance to trypsin was assessed by western blot analysis and results were quantified by densitometry from n = 3 independent experiments. As a control, mutation of residues in the membrane interaction region, which are not active in fusion (see below, Fig 5) but are not involved in trimer contacts (Y88A and F250A), behave like wild type in this assay. The statistical evaluation of each data point was performed in relation to the wild type Gc treated at pH 5.5. ***, P < 0.00025; **, P < 0.0025; *, P < 0.025; ns, not significant.

An important difference with the post-fusion structures of the arbovirus class II proteins is that in the quaternary organization of hantavirus Gc, domain III takes the place occupied in the other trimers by its counterpart from a neighboring protomer. This had been observed previously in the structure of the rubella virus E1 glycoprotein in its post-fusion conformation (Fig 4A), the only other non-arbovirus viral class II protein of known structure [36]. In this altered quaternary organization of the Gc trimer, the strictly conserved glycan chain of Gc at Asn280 in domain II fits snugly into a groove at the subunit interface, with the glycan making a number of inter-chain hydrogen bonds with hydrophilic amino acids at the domain III surface from the adjacent subunit (Fig 4B). Ablation of this glycosylation site in Hantaan virus Gc was shown to give rise to a Gn/Gc glycoprotein complex that was able to reach the cell surface [57], but could not induce syncytium of transfected cells upon low pH treatment [58]. These observations are in line with the added trimer stability provided by the glycan contacts.

The domain III swap is accompanied by the N tail and by strand A_0_ (residues 19–21 in the pre-fusion form), which switches from its parallel interaction with strand B_0_ to run antiparallel (residues 14–19 in the post-fusion form) to C_0_ of the neighboring subunit in the trimer (Fig 2F), thereby augmenting the inner sheet and providing an extensive pattern of inter-subunit main chain hydrogen bonds. Other inter-chain interactions between strictly conserved residues involving the Gc N tail include hydrogen bonds and salt bridges between His14 and Asp174 in β-strand G_0_ and between Asp11 and His399 in domain III, as well as Trp9 packing against Pro321 and disulfide 9 (Cys322-Cys352) (Fig 4C), also in domain III. The domain I/III linker (residues 310–321) runs along the A_0_ strand, making several antiparallel β-sheet interactions with it (Fig 4C). At the very N-terminal end, residues 1 through 5 (which are variable in sequence across the hantaviruses, Fig 1D) project into solvent and are disordered.

As the N tail is not present in the other class II proteins, but in hantaviruses a number of its residues are strictly conserved (Fig 1D) and are seen in the structure to make a network of interactions, we turned to the Andes virus system to functionally test some of these residues. We chose to substitute Asp11, Thr12 and His14 by alanine, and also His14 by tyrosine to see if a bigger side chain could functionally substitute for histidine. These mutants were all expressed correctly and reached the cell surface (S5 Fig), but syncytium formation and cell entry by the corresponding pseudotyped SIV particles was abrogated (Fig 4D and 4E). In order to further explore whether trimerisation of the mutants is impaired, we made virion-like particles (VLPs) of Andes virus [60] harboring the mutations D11A and H14Y in Gc. We harvested VLPs from cells transfected with wild type Gn/Gc or with wild type Gn and the mutant Gc, which secreted VLPs at similar levels (S5 Fig). We analyzed the VLPs after low pH treatment followed by detergent solubilization for Gc trimer formation by sedimentation on a sucrose gradient. The amount of mutant Gc trimer formation was similar to the wild type VLPs (Fig 4F), indicating that trimerization is not impaired.

We therefore analyzed the stability of the resulting acid-induced mutant Gc trimers by trypsin digestion of the VLPs. We observed that, contrary to wild type Gc, the low pH treated D11A and H14Y mutants were not resistant to proteolytic degradation (Fig 4G). In parallel, we found that the alanine substitution of Tyr88, Trp115 or Phe250, which are located at the tip of Gc domain II and which are also impaired in fusion (see below) but for which the side chains are not involved in inter-protomer contacts in the trimer, resulted in trypsin-resistant trimers as wild type Gc, serving as a positive control. These data indicate that the inter-subunit interactions of the Gc N tail are important to confer sufficient stability of the Gc post-fusion trimer in order to be fusion active.

### The membrane interacting region of the Gc trimer

**Fig 5:**
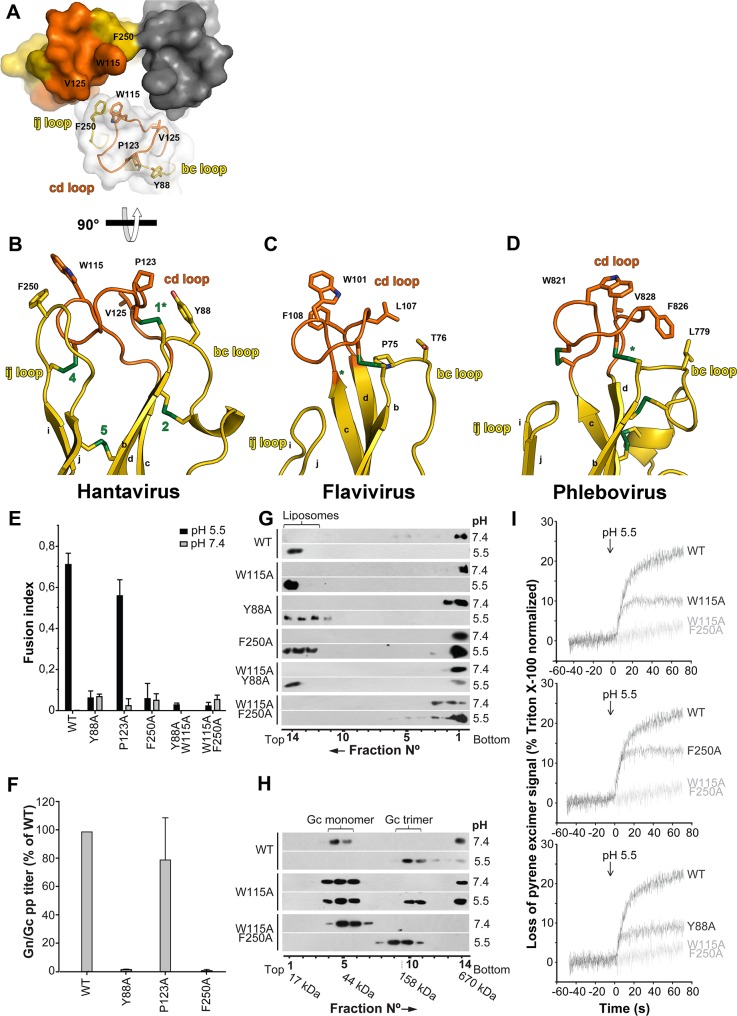
The Gc fusion loops. A) Surface representation of the tip of HNTV domain II viewed down the three-fold axis from the membrane. Two of the subunits are shown in solid surface representation and the third was rendered semi-transparent to visualize the polypeptide chain and selected side chains. B) Orthogonal view of one protomer, with the same residues and loops labeled. C) For comparison, the same representation of flavivirus E (Dengue 2, 1OK8; [53]) and D) phlebovirus Gc (RVFV, 4HJ1; [20]) fusion loop region. Note that in hantavirus Gc, the *ij* loop is longer and projects Phe250 to the membrane, whereas in flaviviruses and phleboviruses, it is not the case (the same holds true for alphaviruses, not depicted here). Similarly, the *bc* loop projects Tyr88 towards the membrane. E) Fusion activity of cells expressing Andes virus wild type Gn and wild type or mutant Gc at neutral or acid pH. Syncytia formation was quantified by counting cells and nuclei using three-color fluorescence microscopy (S6 Fig) and results from at least n = 2 experiments were averaged. F) Entry into Vero E6 cells viral particles pseudotyped with Andes virus spikes with wild type Gn and wild type or indicated mutant Gc (Gn/Gc pp; as in Figs 3D and 4E; see the S7 Fig for cell cytometry quantification of reporter GFP production). G) Liposome co-flotation assay to visualize acid-induced membrane interaction of wild type and mutant Andes virus VLPs. VLPs were incubated with liposomes labeled with a fluorescent dye (DPH, see Materials and Methods) at pH 7.4 or 5.5. Fractions of the step gradient sedimentation were examined for the presence of Gc by western blot and liposomes by fluorescence. H) Trimer formation by wild type and mutant Gc. Sucrose sedimentation of VLPs incubated at pH 7.4 or 5.5 and detection of Gc by western blot analysis. I) Acid-induced lipid mixing kinetics with liposomes of VLPs including Gn and wild type or mutant Gc. The decrease of pyrene-excimer fluorescence intensity was detected upon low pH incubation of pyrene-labeled VLPs with liposomes as a function of time. Results are representative for at least n = 2 independent experiments.

The hantavirus Gc *ij* loop is longer than the corresponding loop in standard class II fusion proteins, and it places Phe250 at the tip of the trimer, along with Trp115 and Pro123 in the *cd* loop and Tyr88 in the *bc* loop (Fig 5A). To understand whether these residues are involved in the functional targeting of the host cell membrane, we performed alanine substitutions and examined the corresponding mutants in the context of Andes hantavirus with the tools described above. We examined these mutants alongside the W115A mutant characterized previously [18]. As W115A, the Y88A, P123A and F250A mutants were properly expressed and the corresponding Gn/Gc complexes trafficked to the plasma membrane of transfected cells (S5 Fig).

Except for the P123A mutant, which behaved as wild type and served a positive control, the Y88A and F250A mutants were impaired in syncytia formation, and did not support entry of the pseudotyped particles into cells (Fig 5E and 5F), similar to the previous results with the W115A mutant. We also analyzed the interaction of VLPs generated with the same mutants with fluorescently labeled liposomes using a sucrose gradient. When VLPs and liposomes incubated at pH 7 were run on the gradient, the liposomes were found by fluorescence floating on top of the gradient, while Gc was recovered from the bottom fractions (Fig 5G). But when the VLPs were incubated with liposomes at pH 5.5, each of the single mutants was recovered in the top fractions of the gradient, as was wild type (Fig 5G), indicating that substitution by alanine of a single residue at the tip of domain II was not sufficient to abolish the interaction with membranes necessary to float with the liposomes.

We also tested VLPs containing double mutations (which produced VLPs in similar yields as wild type, S5 Fig): whereas Y88A/W115A still partially floated with the liposomes, W115A/F250A remained in the bottom fractions at acidic pH. But neither of them mediated low pH-induced syncytia formation, as expected from the single substitution mutants (Fig 5E). In order to identify the actual step at which substitution of W115A and F250A block the membrane fusion process, we tested whether these single or double mutants still underwent acid-induced Gc trimerization, since it was shown previously that Gc can trimerize in the absence of membrane insertion [46]. As expected, sedimentation in a sucrose gradient of acid-treated VLPs including Gc mutants W115A and W115A/F250A revealed that, independently of the membrane inserting activity, these mutants underwent homotrimerization as did wild type Gc (Fig 5H). This result is in line with the structure of the post-fusion trimer, in which the side chains of these residues are not involved in trimer contacts but are exposed at the membrane-interacting side of the trimer (Fig 5A).

To further investigate the stage at which fusion is blocked with these mutants, we incubated pyrene-labeled VLPs [61] bearing wild type Gc or the mutants with liposomes, in order to monitor lipid mixing. Acidification resulted in decrease of the fluorescence of the pyrene excimer within 20 sec in the case of wild type Gc, reflecting lipid mixing (Fig 5I) during fusion. In contrast, the VLPs carrying the Gc double mutant W115A/F250A, which do not insert into target membranes as monitored by liposome flotation, displayed no signal for lipid mixing, corroborating the assay (Fig 5I). When we ran this experiment with VLPs containing the Gc fusion inactive single substitution mutants Y88A, W115A or F250A, we could still detect lipid mixing upon low pH incubation with the liposomes (Fig 5I), indicating that these mutants led to an incomplete fusion process, most likely arrested at the hemifusion stage, as they do not induce full fusion (Fig 5E and 5F). Indeed, if only the labeled lipids of the outer leaflet become diluted during hemifusion, then the expectation is to obtain a lower lipid mixing signal, as observed–, provided that there is no lipid flipping from inner to outer leaflets of the membrane during the time frame of the experiment. The fact that the mutants did not induce full fusion (Fig 5E and 5F) indicates that the observed lipid mixing was due to hemifusion with negligible lipid flipping under the conditions of the experiment.

These results therefore indicate that the Gc single mutants do not insert stably enough into the membrane to induce full fusion, but they still can induce lipid mixing. We conclude from these results that the longer *ij* loop observed in hantavirus Gc, as well as the *bc* loop with Tyr88 projecting into the membrane, have functional implications in engaging the target membrane such that full fusion can proceed. In contrast to the arbovirus class II proteins, which appear to have a single fusion loop–the *cd* loop–in hantaviruses the membrane interacting surface is multipartite.

### Conservation of the hantavirus Gc disulfide bonds pattern across the *Bunyaviridae*

**Fig 6:**
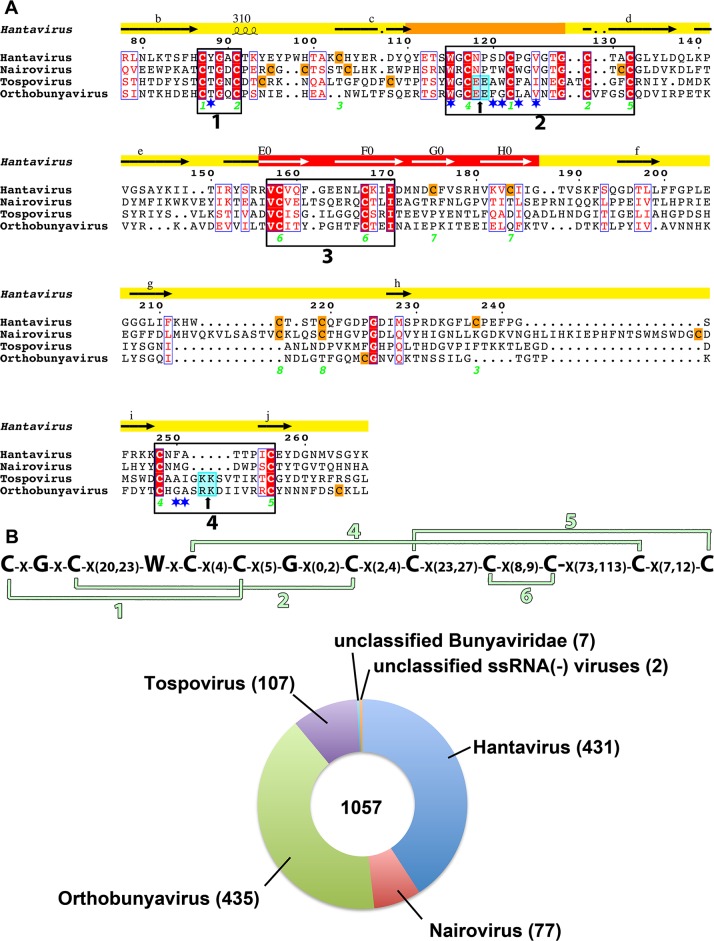
A common tertiary structure motif in Gc from four bunyavirus genera. A) Multiple Gc amino acid sequence alignment from one representative each of the *Hantavirus* (HNTV, P08668.1), *Tospovirus* (TSWV, NP_049359.1), *Orthobunyavirus* (BUNV, NP_047212.1), and *Nairovirus* (CCHFV, AAK52743.1) genera. The secondary structural elements of Hantaan virus Gc are shown in the upper line (as in Fig 1D), the hantavirus disulfide bonds are labeled, and cysteines not conserved across genera are in an orange background. The residues exposed at the membrane-interacting end of domain II in hantavirus Gc are marked with a blue star under the alignment. The four boxes mark clusters of high conservation, which are very informative when analyzed by pairs, *Hanta*-*Nairovirus* and *Tospo*-*Orthobunyavirus* genera, which are closer to each other than to the others (see the S8 Fig). A cyan background within boxes 2 and 4 (marked also by a small black arrow underneath) highlights amino acids with complementary electrostatic charges predicted to come into contact at the membrane interacting surface, between disulfides 4 and 5, which connect *cd* and *ij* loops. B) The motif obtained from the alignment in A (top line) was run in Prosite to scan the SwissProt and trEMBL databases, and the taxonomic distribution of the matched entries are provided in the colored pie chart. The 1057 entries found encompass all of the known viruses within the four genera, plus some that are “unclassified” by the server. Searching the literature shows that the 7 viruses given as “unclassified *Bunyaviridae”* have been assigned to one of the genera (three orthobunyaviruses: Buffalo Creek, Mapputa and Marpik viruses [62]; one tospovirus [63]; and three nairoviruses: Sanxia Water Strider, Shayang Spider and Xinzhou Spider viruses [64]. The two “unclassified ssRNA negative-polarity viruses” are Wuhan Louse Fly virus and Wuhan insect virus 2, which were reported in a recent metagenomic analysis [64] and remain unclassified.

Amino acid sequence alignment of hantavirus Gc with its counterparts from viruses of the various genera of the *Bunyaviridae* family allowed the identification of a motif that systematically identifies Gc glycoproteins from four out of the five genera, leaving out the phleboviruses. The alignment used to extract this motif is displayed in Fig 6, and includes four of the disulfide bonds that stabilize the tip of domain II as well as one of the disulfides in domain I (the one stapling together β-strands E_0_ and F_0_). This alignment allows the prediction of the connectivity of the additional cysteines in the other genera (S8 Fig).

Two features appear important: as hantavirus Gc, the other genera also have N-terminal extensions, which are quite large (as in orthobunyavirus Gc), and lack the N-terminal disulfide bond stapling β-strand A_0_ and C_0_ as in the other class II fusion proteins featuring an A_0_ strand (i.e., phlebovirus Gc, flavivirus E and the cellular fusion protein EFF-1). Such a disulfide bond would be incompatible with the rearrangements of strand A_0_ that are necessary to have a swapped domain III in the post-fusion form, and one prediction therefore is that the phlebovirus Gc will have the “standard” class II quaternary arrangement, whereas in all the other genera the post-fusion form of Gc is likely to feature a swapped domain III.

Similarly, the *ij* loop is also longer in the *Nairovirus*, O*rthobunyavirus* and *Tospovirus* genera than in the standard class II proteins, and is likely to contribute to the membrane interacting surface (see Fig 5A, 5B and 5C). In contrast, the carboxylate—carboxylic acid hydrogen bond in strand *c*, which structures the tip of domain II, appears as a hantavirus-specific feature, as these residues are not conserved across these bunyavirus genera.

## Discussion

Membrane fusion is a critical step in entry for any enveloped virus, and in hantaviruses it is mediated by the highly conserved glycoprotein Gc. Because of this conservation, the features identified in the structures of Hantaan virus Gc in its pre- and post-fusion forms can be applied to all members of the *Hantavirus* genus. We have taken advantage of this conservation to use the molecular tools developed to test the glycoproteins of Andes virus for function. By combining structural and functional data, one important aspect that we have discovered is the multi-partite nature of the membrane interaction surface of hantavirus Gc as well as the key role played by the N tail for fusion. These results set hantavirus Gc—and by extension, also Gc from the three more closely related genera in the *Bunyaviridae* family, see below–apart from the more standard class II fusion proteins observed in the flaviviruses and phleboviruses. The latter have an A_0_ strand (contrary to alphavirus E1, which lacks strand A_0_) but it is locked by a disulfide bond to the C_0_ strand of the same polypeptide chain, prohibiting a conformational rearrangement similar to the domain III swap, which is accompanied by the N tail (Fig 2F).

The various loops interacting with the target membrane in hantavirus Gc are reminiscent of the bi-partite membrane contacting region of class III fusion proteins [65], with two fusion loops—which may also require elements from the C-terminal, membrane proximal region to engage the target membranes and induce full fusion. A more extensive membrane binding region was previously observed in the class II fusion protein E1 of rubella virus, which features an insertion within the *cd* loop to make two short α-helices and an additional β-strand (*c’*) running parallel to strand *c*, such that the *bdc* β-sheet at the tip of domain II becomes a four-stranded *bdcc’* β-sheet [36]. This results in two fusion loops, *cc’* and *c’d*, projecting toward the target membrane, and a Ca^2+^ site in between the two loops that is essential for function [66]. An analogous role appears to be played by Asn118 in hantavirus Gc, by bridging two loops in the membrane interacting region (Fig 2E). A further similarity with the rubella virus post-fusion E1 trimer is the swapped domain III, although in E1 there is neither A_0_ strand nor N tail to accompany this conformational change, and the transition is not yet understood in the absence of a structure of E1 in its pre-fusion form.

The mechanistic implications derived from the structures of the hantavirus Gc can be extended to other bunyaviruses, further broadening the scope of this work. Indeed, our comparison of hantavirus Gc with its counterparts of the other genera indicates that the hantavirus and nairovirus Gc proteins are closer to each other than they are to Gc from other bunyaviruses, and that Asn118, which plays a key structuring role within the *cd* loop and the interaction with the *ij* loop (Fig 2E) is conserved across the two genera (Fig 6 and S8 Fig). We also note the conservation in nairoviruses of Trp9 and His14 of the N tail, which are involved in the network of interactions illustrated in Fig 4C, supporting the notion that a similar domain III swap may occur in nairoviruses as well. As nairoviruses require the additional cleavage of preGc into mature Gc at the N-terminal end, at an “RKPL” site corresponding to a non-standard subtilase SKI-1 like protease [67], it is possible that the cleavage is necessary to release the N tail such that the fusogenic conformational change can take place (the RKPL sequence is about 40 residues upstream the first residue displayed in the alignment of the S8A Fig).

The sequence conservation pattern further shows that orthobunyavirus and tospovirus Gc proteins are also closer to each other than they are to the others (S8B Fig), and feature an insertion in the *ij* loop, which becomes even longer (Fig 6). The amino acid alignment indicates that a dibasic motif downstream the disulphide 4 in the *ij* loop (highlighted in a cyan background in Fig 6) will face an acidic “EE” motif in the *cd* loop (highlighted in the same Figure) at the location of Asn118 in hanta- and nairoviruses, suggesting that a different set of polar/electrostatic interactions may replace the network formed by Asn118 at the tip of domain II in Gc of these two genera.

Our results on hantavirus Gc therefore reveal a number of unanticipated aspects of the bunyaviruses in general. In particular, they introduce an evolutionary hierarchy for the five genera based on the Gc gene, which appear to display a deep branching site between phleboviruses and the others, the latter then splitting into two branches each, giving rise to hantaviruses and nairoviruses on the one hand, and to orthobunyaviruses and tospoviruses on the other, with mechanistic similarities for the membrane fusion process accompanying this diversification. We thus propose to group these fusion proteins as a sub-class within class II, having by specificity the fact of presenting a multipartite membrane targeting region, together with an N tail involved in the fusogenic conformational rearrangement.

Additional structure-function studies on the Gc orthologs from each of these branches will further nail down the practical implications of the identified evolutionary trends, opening the possibility of unveiling common vulnerability sites on Gc to be targeted by broad-spectrum anti-bunyavirus compounds to help treat patients against these deadly pathogens.

## Materials and Methods

### Protein expression and purification

In order to obtain milligrams amount of soluble Hantaan virus Gc, we inserted a synthetic gene codon-optimized for expression in *Drosophila* cells, into a modified pMT/BiP plasmid (Invitrogen). This initial construct, termed pMT-Gn/rGc, included the full length Gn followed by the Gc ectodomain, i.e. lacking the transmembrane and cytoplasmatic domains. To help in the purification we included two strep-tag sequences separated by a (GGGS)_3_ linker and preceded by an enterokinase cleave site. As this construct produced only small amounts of soluble rGc, most likely because it is retained within the cells, either by Gn and/or because the fusion loop of Gc interacts with cellular membranes, we removed Gn from the construct to make pMT-rGc. As mentioned in the main text, we also introduced the W115H mutation in this plasmid (pMT-rGc-W115H). Similarly, scFv A5, identified as binding to rGc by screening the Griffin library using helper phage KM13 [68], was cloned into the modified pMT/BiP plasmid followed by a double strep-tag (pMT-scFvA5). These plasmids were used separately to obtain stable transfectants of Drosophila S2 cells together with the pCoPuro plasmid (ratio 1:20) for puromycin selection. The stable cell lines were selected and maintained in serum-free Insect-Xpress medium containing 7 μg/ml puromycin. Cultures of 1–3 liters were grown in spinner flasks in Insect-Xpress medium supplemented with 1% penicillin/streptomycin antibiotics to about 1 x 10^7^ cells/mL, and the protein expression was induced with 4 μM CdCl_2_. After 5 days, the S2 media supernatant was concentrated to 40 ml and supplemented with 10 μg/mL avidin and 0.1M Tris-HCl pH 8.0, centrifuged 30 minutes at 20,000 g and purified by strep-tactin affinity chromatography and gel filtration. The yields were about 5–10 mg/L for rGc and rGc-W115H and 10–15 mg/L for scFv A5.

For crystallization, rGc was incubated on ice for 30 minutes with a 1.2 molar excess of scFv A5 with the pH adjusted to 8.5 by adding TrisHCl pH 8.5 to a final concentration of 100 mM. The mixture was digested with trypsin (mass ratio of 1:100), for 1 hour at 37°C, stopping the reaction by adding 1 mM of PMSF and cooling on ice. The digest was loaded to a gel filtration Superdex 75 16/60 column in 10 mM Tris HCl pH 8.0, 150 mM NaCl, and the fractions of the peak corresponding to the complex were pooled and concentrated in the same buffer for crystallization trials.

The rGc-W115H construct was used to obtain the post-fusion form without adding detergent, as explained in the main text. Digestion with enterokinase (New England Biolabs) after Strep-tactin affinity purification to remove the Strep-tag for crystallization showed an immediate cleavage but overtime a second, shorter resistant fragment accumulated overnight at 4°C. Only this second enterokinase resistant fragment, in which rGc appeared to lose the stem region, resulted in crystals. Inspection of the stem region indeed indicates several lysine residues where enterokinase could cleave. In the final protocol, the enterokinase treatment was allowed to proceed overnight at 4°C, and then the digest was submitted to gel filtration on a Superdex 200 16/60 column in 10 mM Tris HCl pH 8.0, 150 mM NaCl. The protein was then buffer exchanged and concentrated in 20 mM Bis-Tris pH 6.1, 150 mM NaCl, using a Vivaspin centricon, to a final concentration of 5–10 mg/ml.

### Crystallization and structure determination

We determined the structure of the rGc/scFv A5 complex by a combination of molecular replacement (MR) and single-wavelength anomalous dispersion (SAD). Native crystals were grown in 60 mM Na-HEPES pH 7.5, 40 mM hexamine cobalt chloride salt, 13.5% (w/v) PEG 4K, 7.4% (v/v) 2-propanol, and 1% (v/v) glycerol and cryo-protected in the same solution supplemented with 22% (v/v) PEG 400. The crystals used for the heavy atom derivative were grown in presence of 60 mM Na-HEPES pH 7.5, 20 mM hexamine cobalt chloride salt, 13% (w/v) PEG 4K, 7.4% (v/v) 2-propanol, and 1% (v/v) glycerol. They were subsequently soaked for 18h in mother liquor supplemented with 2 mM samarium and cryo-cooled using 22% (v/v) PEG 400 as cryo-protectant before plunging into liquid nitrogen. In order to optimize the anomalous signal we collected a highly redundant dataset (S1 Table), merging three independent datasets collected from a single crystal using a kappa goniometer (kappa = 10°, 0°, -10°) using an “inverse beam” strategy. The final SAD dataset could be processed up to a resolution of 3.65 Å and showed a usable (CCanom > 0.3) anomalous signal up to 4.5 Å.

To determine the phases, we first modelled an scFvA5 molecule using the RosettaAntibody3 program [69] through the ROSIE interface [70]. We then used the resulting model as MR template for PHASER [71] with the native dataset at 3 Å resolution. We obtained a single solution with a translation function Z-score of 7.4 in which the scFv molecules are packed together back to back around a two-fold axis, with the CDR loops exposed to solvent, leaving enough space to accommodate one Gc molecule. The initial MR phases were used to identify a set of heavy atom sites in the Sm-SAD dataset using the HySS (Hybrid Substructure Search) module of the PHENIX package [72], and we used them to calculate SAD phases with PHASER. The combined MR and SAD phases were improved by density modification using RESOLVE [73]. All these steps were done automatically using the AutoSol wizard [74] in PHENIX. The final map was of enough quality to start manual model building, and the extended and corrected model was used as input model in a new MR-SAD cycle. With this iterative procedure we built a model that comprises full domains I and III, and a large part of domain II. We used domains I and III as MR templates to determine the structure of the rGc-W115H trimer at 1.6 Å resolution and build a complete model using this dataset. We then used the structure of the high-resolution post-fusion form as a guide to finish the model building and to generate restraints for refinement of the complex rGc/scFvA5.

The crystals of the rGc-W105H trimer were grown in 100 mM Na-MES pH 6.5, 10.8% (w/v) PEG 8K, and 7% (v/v) glycerol and were cryo-protected in the same solution supplemented with 20% (v/v) glycerol. As described above, the structure was solved by MR using the partial models of domains I and III from the rGc/scFvA5 crystals. We also identified conditions of rGc-W105H trimer crystal growth in the presence of 500 mM KCl, which showed no density for the fusion loop region. We then carried out a detailed study of the effect of KCl by growing crystals of rGc-W105H in the presence of increasing concentrations of KCl. We collected datasets in the presence of 100 mM KCl, (resolution 1.8 Å), 200 mM KCl (1.7 Å), 300 mM KCl (1.6 Å), 500 mM (1.5 Å), and 600 mM KCl (1.4 Å), where the values in parentheses are the resolution limits in each case. The structures of these crystals were determined by molecular replacement using as a model rGc-W105H. We refined the structures using phenix.refine and the final model validated with Molprobity [75] and CheckMyMetal [76]. The statistics of all crystals and refinement is provided in the S1 Table.

### Liposome preparation

Liposomes were prepared fresh each time by the freeze-thaw and extrusion method [77]. DOPC (1,2-dioleoyl-sn-glycero-3-phosphocholine), DOPE (1,2-dioleoyl-sn-glycero-3-phosphoethanolamine), sphingomyelin (from bovine brain), cholesterol (from ovine wool), PC (phosphocholine, from egg yolk) and PE (phosphoethanolamine, prepared from egg phosphatidylcholine by transphosphatidylation) were purchased from Avanti Polar Lipids. Type 1, 2, 3 and 4 liposomes were made using DOPC alone, DOPC/cholesterol (1/1), DOPC/DOPE/sphingomyelin/cholesterol (1/1/1/3) and PC/PE/sphingomyelin/cholesterol (1/1/1/1.5), respectively.

### Electron microscopy experiments

Samples of rGc were negatively stained with phosphotungstic acid and screened with a Tecnai G2 Spirit Biotwin microscope 5 (FEI, USA) operating at an accelerating voltage of 120 kV. To obtain the rGc/liposomes pictures, 200 ng of rGc were added to 10 ul of a solution of 1 mM of type 3 liposomes in 20 mM MES pH 5.5, 150 mM NaCl (MN 5.5). After 1 minute incubation at room temperature, the sample was spotted onto carbon coated glow discharged grids, contrasted with 2% phosphotungstic acid and screened in a TecnaiG2 Spirit Biotwin microscope operating at an accelerating voltage of 120 kV. Images were recorded using a 4Kx4K camera Eagle (FEI, USA) and the TIA software (FEI, USA).

### Surface Plasmon Resonance (SPR) assays

All experiments were performed on a Biacore T200 instrument (GEHealthcare) equilibrated at 25°C in two different running buffers: PBS pH 7.4, and MN pH 5.5 (20 mM MES pH 5.5, 150 mM NaCl).

#### Inhibition of the interaction of rGc with liposomes by scFv A5

Liposomes of different concentrations (500 μM in PBS) were captured on the four flow cells of an L1 sensor chip (GE Healthcare) for 5 minutes at a flow rate of 5 μl/min, reaching a density of 7500–8000 resonance units (RU). The surface was washed with 20 mM NaOH for 1 minute and saturated with 0.2 mg/ml of BSA for 5 minutes. rGc (500 nM) alone or mixed with scFv A5 or the control scFv B (200, 1000, and 5000 nM) was then injected for 1 minute at 50 μl/min over the liposome surface. Blank injections of scFvs alone or PBS were performed in parallel. Finally, the surfaces were regenerated with two 2-minutes injections of 20 mM CHAPS and one 1-minute injection of 0.1% SDS.

#### Interaction of Gc with scFv A5 and B

The scFvs were covalently immobilized by amine coupling on two flow cells of a CMD200m sensor chip (Xantec), reaching densities of 900 RU for A5 and 550 RU for B. rGc (39–5000 nM) was injected over the scFv surfaces for 3 minutes at 50 μl/min followed by 5 minutes of dissociation in each of the two running buffers (PBS and MN 5.5). Surfaces were regenerated with a 30 seconds injection of 0.01% SDS. Signals measured on the scFv B surface were used as references and subtracted from those measured on the scFv A5 surface. The association/dissociation profiles were analysed using the Bioevaluation software yielding k_on_, k_off_ and K_D_ values.

### Design of Gc mutants and expression in transfected cells

We used the expression plasmid pI.18/GPC for the expression of the full length GPC coding region (encompassing both Gn and Gc in the M genomic segment) of Andes virus strain CHI-7913 [56]. Site-directed mutations were generated by DNA synthesis and sub-cloning into pI.18/GPC using intrinsic restriction sites. For expression, 293FT cells (Invitrogen) (3.6×10^6^) grown in 100 mm plates were calcium-transfected with 8–20 μg of DNA and 48 h later, cell surface proteins were labelled with biotin in order to separate the biotinylated (surface proteins) from non-biotinylated (intracellular proteins) fractions using a cell surface protein isolation kit (Pierce). For protein detection by western blot, primary antibodies anti-Gc monoclonal antibody (MAb) 2H4/F6 [78] or anti-β-actin MAb (Sigma) were used at 1:2,500 and subsequently detected with an anti-mouse immunoglobulin horseradish peroxidase conjugate (Thermo Fisher Scientific) 1:5,000 and a chemiluminescent substrate (SuperSignal WestPico, Pierce).

### Cell-cell fusion experiments

This assay was performed as previously described [18]. Briefly, Vero E6 cells (ATCC) seeded into 16 well chamber slides were transfected with the pI.18/GPC wt or the different mutant constructs using lipofectamine 2000 (Invitrogen). The DNA amounts were adjusted to obtain similar levels of Gc at the cell surface; the cells were accordingly transfected with plasmid DNA ranging between 0.5–1.5 μg. 48 h later, the cells were incubated in E-MEM (pH 5.5) at 37°C for 5 min, subsequently washed, and the incubation continued for 3 h at 37°C in E-MEM (pH 7.2). The cell cytoplasm was then stained for one hour with 1 μM of CellTracker CMFDA (Molecular Probes) and cells were next fixed for 20 min with 4% paraformaldehyde. For immunofluorescence labelling, cells were permeabilized with 0.1% Triton X-100 in PBS and Gc labelled using the MAb 2H4/F6 1:500 and secondary antibody anti-mouse immunoglobulin conjugated to Alexa555 1:500 (Invitrogen). Finally, nuclei were stained for 5 min with DAPI 1 ng/μL and samples examined under a fluorescence microscope (BMAX51, Olympus). The fusion index of Gc-expressing cells was calculated using the formula: 1- [number of cells/number of nuclei]. Approximately 200 nuclei per field were counted (20X magnification) and five fields used to calculate the fusion index for each sample (n = 2) of two independent experiments.

### Glycoprotein assembly into VLPs

VLPs were harvested from supernatants of 293FT cells transfected with the pI.18/GPC wt or the different mutant constructs at 48 h post-transfection as previously established [60]. Subsequently, VLPs were concentrated by ultracentrifugation for one hour at 135,000 g and detected by reducing SDS PAGE and western blot using anti-Gc MAb as described above. The presence of VLPs was further corroborated by negative-stain electron microscopy using phosphotungstic acid 2% pH 7.4 (FEI Tecnai 12 Transmission Electron Microscope, Philips).

### Production of pseudotyped SIV vectors and transduction

Simian immunodeficiency virus (SIV) vectors pseudotyped with ANDV Gn and wild type or mutant Gc were prepared as previously described [56]. Briefly, 293FT were transfected with the following plasmids: pSIV3+ [79], pGAE1.0 [80] (kindly provided by Jean-Luc Darlix, INSERM, ENS-Lyon, France) and pI.18/GPC wild type or the different mutant constructs. At 72 h post-transfection, pseudotyped vectors released into the supernatant were harvested, concentrated by ultracentrifugation at 135,000 g and used to transduce Vero E6 cells (ATCC, CR-1586). 72 h post-inoculation, cells were trypsinized and GFP expression assessed by flow cytometry (FACScan, Becton Dickinson). Transduction titers were calculated using the percentage of GFP positive cells, counting at least 10,000 cells of each condition.

### Liposome-coflotation experiments

The coflotation of viral particles with liposomes was performed as previously established [46]. Briefly, VLPs or GPC-pseudotyped SIV vectors prepared from wild type or mutant pI.18/GPC construct were incubated with 200 mM 1,6-diphenyl-1,3,5-hexatriene (DPH)-labelled liposomes (Type 4) for 30 min at pH 7.4 or 5.5. The VLP-liposome mixture was then added to the bottom and adjusted to 25% (w/v) sucrose. Additional sucrose steps of 15% and 5% were then over-layered. After centrifugation for 2 h at 300,000 g, liposomes were detected by the fluorescence emission of DPH (λex = 230 nm; λem = 320 nm) and VLPs by western blot using anti-Gc MAb 2H4/F6.

### Gc homotrimerization experiments

Acid-induced Gc homotrimerization was tested as established before [46]. VLPs or GPC-pseudotyped SIV vectors prepared from wt or mutant pI.18/GPC constructs were incubated for 30 min at 37°C at the indicated pHs to allow multimerization changes. Next, Triton X-100 1% (v/v) was added to allow the extraction of the membrane glycoproteins from the viral particle. The extracted glycoproteins were then added to the top of a sucrose gradient (7–15%; w/v) and centrifuged at 150,000 g for 16 h. Finally, fractions were collected and the presence of Gc was analyzed by western blot using MAbs anti-Gc 2H4/F6.

### Trypsin resistance

The stability of the Gc homotrimer was studied by analyzing its resistance to trypsin digestion as determined before [46]. VLPs were incubated at pH 5.5 for 30 min at 37°C to allow Gc multimerization changes or pH 7.4 as a digestion control. Next, the VLPs were digested with 500 μg/ml of TCPK trypsin (Sigma) for 30 min, stopping the reaction by the addition of sample buffer and heating to 95°C for 10 min. The extent of Gc digestion was determined by western blot, using an anti-Gc MAb. The trypsin resistance of wild type and mutant Gc was quantified by dividing the densitometry values of the digested Gc signal by the undigested Gc assay input control for each mutant, using the Fiji software [81]. The average value and standard derivation of 3 experiments was calculated and a Student’s t test was performed for statistical evaluation: ***, P < 0.00025; **, P < 0.0025; *, P < 0.025.

### Lipid mixing assay

For the lipid mixing assay VLPs were metabolically pyrene-labeled by supplementing the producing cell’s media with 25 μg/ml of 1-pyrenehexadecanoic acid (Molecular Probes). Labeled VLPs were mixed with liposomes and lipid mixing was monitored by the decrease in pyrene excimer fluorescence generated by the dilution of the pyrene-labeled phospholipids with the unlabeled phospholipids in the liposome membrane in a continuously stirred fluorimeter cuvette at 37°C. Fluorescence was recorded continuously at 480 nm using a Varian Eclypse Fluorescence Spectrophotometer (Agilent Technologies) at an excitation wavelength of 343 nm using a 10-nm slit width for excitation and emission. After a stable base line was established, the pH of the solution was lowered to 5.5 for reaction initiation (time = 0). The base line excimer value was defined as 0% lipid mixing and the maximal extent of excimer dilution was defined by the addition of detergent Triton X-100 after lipid mixing of each condition concluded.

### Data Availability Statement

All relevant data are within the paper and its Supporting Information files. The coordinate files of the structures described in this manuscript have been submitted to the Protein Data Bank, and the corresponding accession codes are listed in the S1 Table.

## Supporting Information

S1 Fig Recombinant Gc is a monomer and makes a 1:1 complex with scFv A5. A) Size exclusion chromatography profile of Gc at neutral (black) and acidic pH (green) and multi-angle static light scattering (MALS) molecular mass determination of each peak, showing that Gc is a monomer in both cases, although at neutral pH the Stokes radius is larger. B) Size exclusion profile of scFv A5 (magenta), Gc (green), and a mixture of Gc with an excess of scFvA5 (blue line). The experiments here were done in Tris pH 8. scFv A5 alone (magenta curve) elutes as a double peak with corresponding molecular mass difference of 3 kDa, which we interpreted to be due to spontaneous degradation and loss of the of the C-terminal purification tag. Gc runs as a single monomer and the mixture of Gc with an excess of scFvA5 produces a shift of the Gc peak of about 16 kDa. Very likely this shift is lower than the expected 24 kDa because of the heterogeneity of the peak, which clearly overlaps with the uncomplexed Gc peak. C and D) SPR sensorgrams showing the interaction between Gc and scFv A5 at neutral (C) and acid pH (D). The antibody was immobilized on the chip and the measurements were done by flawing Gc at concentrations of 5000, 2500, 1250, 625, 312, 156, 78, and 39 nM.
(JPG)

S2 Fig rGc binds to cholesterol containing liposomes, and binding is inhibited by scFv A5. A) The rows correspond to liposomes of different compositions, as indicated in the right, which were immobilized on an L1 chip. 500 nM of Gc alone (red line) or in combination with increasing amounts (green, blue, and cyan curves) of scFv A5 (first and third column) or a control scFv (scFv “B”, second and fourth columns) were injected at pH 5.5 (first and second columns) and 7.4 (third and fourth column), as indicated in the top line, using as running buffer PBS (pH 7.4) or buffer MN (pH 5.5, see Methods for buffer composition). B) Model of three scFvs A5 interacting with a Gc trimer, showing that the binding mode is compatible with trimer formation but not with trimer insertion into membranes.
(JPG)

S3 Fig Biochemical properties mapped to the Gc surface. Shown are the post-fusion trimer and one of it subunits (i.e., the two foreground subunits were omitted, leaving only the one in the back, to show the buried surface within the trimer). The trimer interface is outlined in green. A) Exposed (left) and buried (right) conserved patches (white). B) Exposed (left) and buried (right) hydrophobic patches (white) and C) electrostatic surface potential. The three-fold axis is indicated in red in all the panels.
(JPG)

S4 Fig The rGc trimer crystallized in the presence of increasing KCl concentrations. The upper panels show the structures of one Gc protomer extracted from the post-fusion trimer colored according the B-factor when crystallized at the indicated KCl concentration. We used the “cartoon putty” option in Pymol [82], in which the radius of the cartoon is proportional to the B factor, to highlight the most mobile regions. The mobility of the fusion loop increases until its electron density is lost in the structure at 300 mM KCl, with a concomitant increase in the resolution to which the crystals diffract. The middle and lower panels compare the polar network of interactions in the protein crystallized at different KCl concentrations to that in the absence of KCl. A curved red arrow in the right-hand panel, middle row, follows the movement of the Glu106 side chain, represented in thicker sticks in this panel, which is concomitant with disordering of the tip. The lower-right panel recapitulates the observed movement of Tyr105 and Glu106 by superposing all the previous panels (See S4 Movie).
(JPG)

S5 Fig Characterization of expression yields and cell localization of Andes virus Gc mutants. Western blot analysis using anti-Gc or anti-β-actin MAbs of different fractions obtained from 293FT cells expressing Gn and wild type or mutant Gc after surface biotinylation. The fractions correspond to the non-biotinylated fraction (intracellular proteins), the biotinylated fraction (surface proteins), or the concentrated supernatant. “Unrelated” indicates the expression of unrelated Gc mutants used as control.
(JPG)

S6 Fig Syncytia formation by wild and mutant Gc. Inmunofluorescence images of Vero E6 cells expressing Gn and wild type or mutant Gc after treatment at pH 5.5. The cell cytoplasm was labelled with 5-chloromethylfluorescein diacetate (CMFDA; green fluorescence), nuclei with DAPI (blue fluorescence) and Gc was detected with anti-Gc antibody (Alexa555; red fluorescence). Cells from a partial microscopy field are shown from a representative experiment. Arrow heads indicate syncytia. (200 x magnification).
(JPG)

S7 Fig Transduction of Vero E6 cells by particles pseudotyped with Gn and wild type or mutant Gc. Flow cytometry gates were set for cells transduced with Mock particles (empty pI.18 vector) and the percentages shown correspond to GFP-positive cells. Data from a representative experiment are shown.
(JPG)

S8 Fig Disulfide conservation pattern across bunyavirus genera: A) Amino acid sequence alignment of Hantaan virus (HNTV, P08668.1) and Crimean-Congo Hemorrhagic Fever Virus (CCHFV, AAK52743.1). The secondary structures of HNTV are indicated above the sequences and the secondary structure predicted by PSIPRED [83, 84] for CCHFV Gc below the alignment. Shown is only the aligned region of CCHFV Gc, which is much longer. The disulfide bond connectivity in HNTV Gc is shown with green numbers and the predicted connectivity in CCHFV is shown with red numbers. The four conserved regions across hantaviruses, nairoviruses, orthobunyaviruses, and tospoviruses are boxed and numbered as in Fig 6. A 5^th^ box indicates a region with conserved orthobunya, tospo and nairovirus cysteine residues, but less so in hantaviruses, spanning the AB loop in domain III. B) Corresponding amino acid sequence alignment of tomato spotted wilt tospovirus (TSWV, NP_049359.1) and Bunyamwera orthobunyavirus (BUNV, NP_047212.1) with the predicted secondary structures above and below the sequences and the predicted disulfide bond connectivity displayed with red and cyan numbers, respectively. C) Cartoon representation of the tip of the HNTV Gc domain II showing the location of the predicted disulfide bonds in CCHFV, BUNV, and TSWV Gc, drawn as cylinders connecting the predicted location of the corresponding cysteine residues color-coded as on the alignment of panels A and B.
(JPG)

S1 Table Crystallographic Statistics of all structures determined. Each column represents a different diffraction data set, as indicated. The “beam line” row indicates, in parenthesis, the synchrotron to which the line belongs (ESRF: European Synchrotron radiation Facility, Grenoble, France; SOLEIL: French Synchrotron Laboratory at St Aubin, France).
(DOCX)

S1 Movie Quality of the electron density map for rGc in its post-fusion form. Electron density maps contoured at 1 sigma level of the region around Glu106 for the low pH rGc structure crystallized in absence of KCl. The electron density around the protein is colored black, and surrounding waters red. The residues involved in the network of hydrogen bonds are labeled.
(MOV)

S2 Movie Quality of the electron density map in the KCL-containing crystals. Electron density maps contoured at 1 sigma level of the region around Glu106 for the low pH rGc structure crystallized in presence of 600 mM KCl. The electron density around the protein and the K+ ion are colored black, and surrounding waters red. The residues involved in the polar network are labeled.
(MOV)

S3 Movie Quality of the electron density map in the crystals of the rGc/A5 complex. Electron density maps contoured at 1 sigma (black) or 0.4 sigma (green) levels of the region around Glu106 for the structure of the neutral pH structure of the rGc/scFvA5 complex. The residues involved in the polar network are labeled.
(MOV)

S4 Movie K^+^ binding alters the conformation of Glu106 on the c strand. The different panels of the S4 Fig were concatenated to create an animation illustrating the structural changes upon K^+^ binding.
(MP4)
