# Supplementary figures and images for: Mechanistic Insight into Bunyavirus-Induced Membrane Fusion from Structure-Function Analyses of the Hantavirus Envelope Glycoprotein Gc
Source: PLoS Pathog. 2016 Oct 26;12(10):e1005813. doi: 10.1371/journal.ppat.1005813 (PMC5082683; doi:10.1371/journal.ppat.1005813)

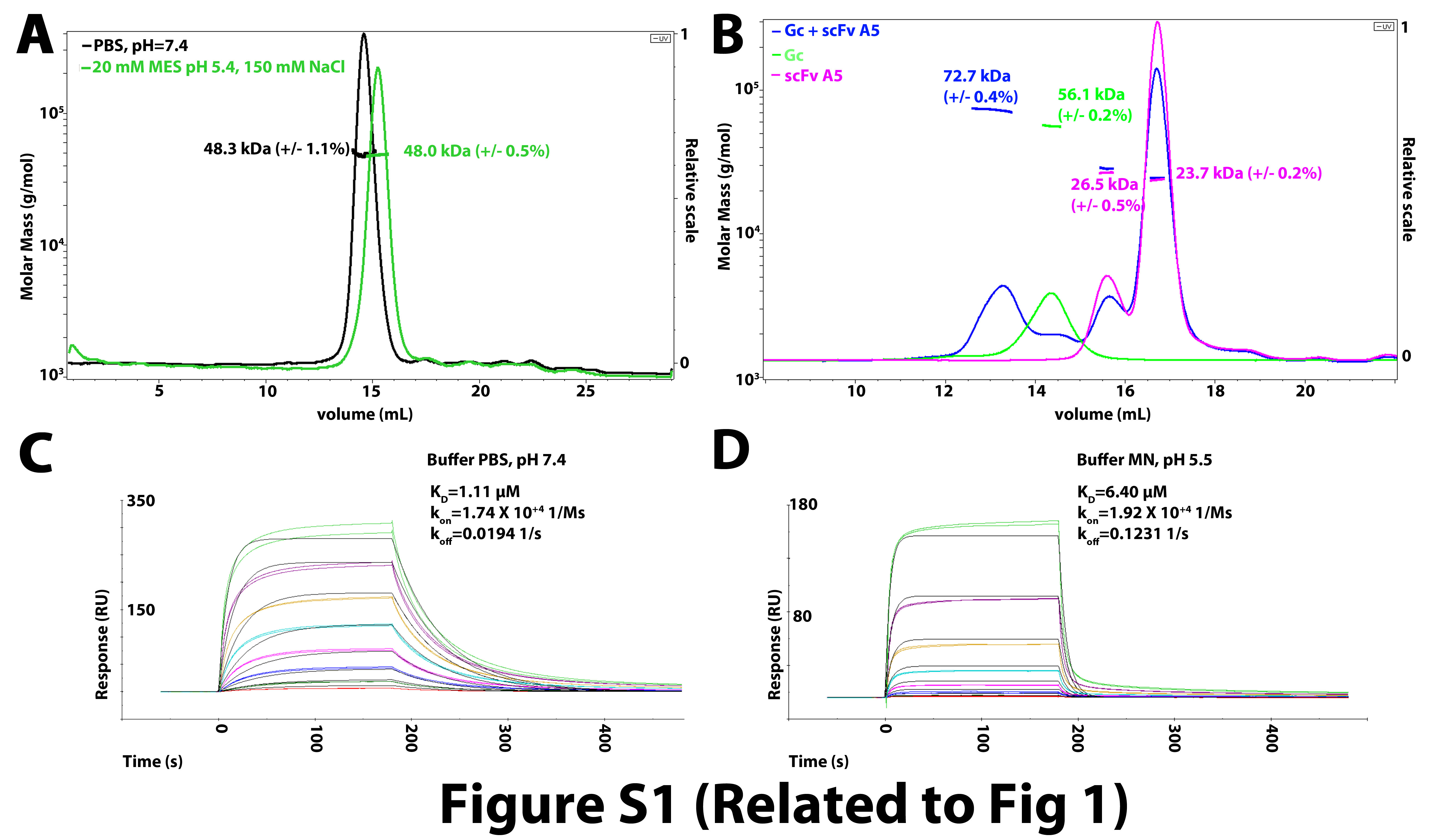

Supplement: S1 Fig — A) Size exclusion chromatography profile of Gc at neutral (black) and acidic pH (green) and multi-angle static light scattering (MALS) molecular mass determination of each peak, showing that Gc is a monomer in both cases, although at neutral pH the Stokes radius is larger. B) Size exclusion profile of scFv A5 (magenta), Gc (green), and a mixture of Gc with an excess of scFvA5 (blue line). The experiments here were done in Tris pH 8. scFv A5 alone (magenta curve) elutes as a double peak with corresponding molecular mass difference of 3 kDa, which we interpreted to be due to spontaneous degradation and loss of the of the C-terminal purification tag. Gc runs as a single monomer and the mixture of Gc with an excess of scFvA5 produces a shift of the Gc peak of about 16 kDa. Very likely this shift is lower than the expected 24 kDa because of the heterogeneity of the peak, which clearly overlaps with the uncomplexed Gc peak. C and D) SPR sensorgrams showing the interaction between Gc and scFv A5 at neutral (C) and acid pH (D). The antibody was immobilized on the chip and the measurements were done by flawing Gc at concentrations of 5000, 2500, 1250, 625, 312, 156, 78, and 39 nM. (JPG) [file ppat.1005813.s001.jpg]

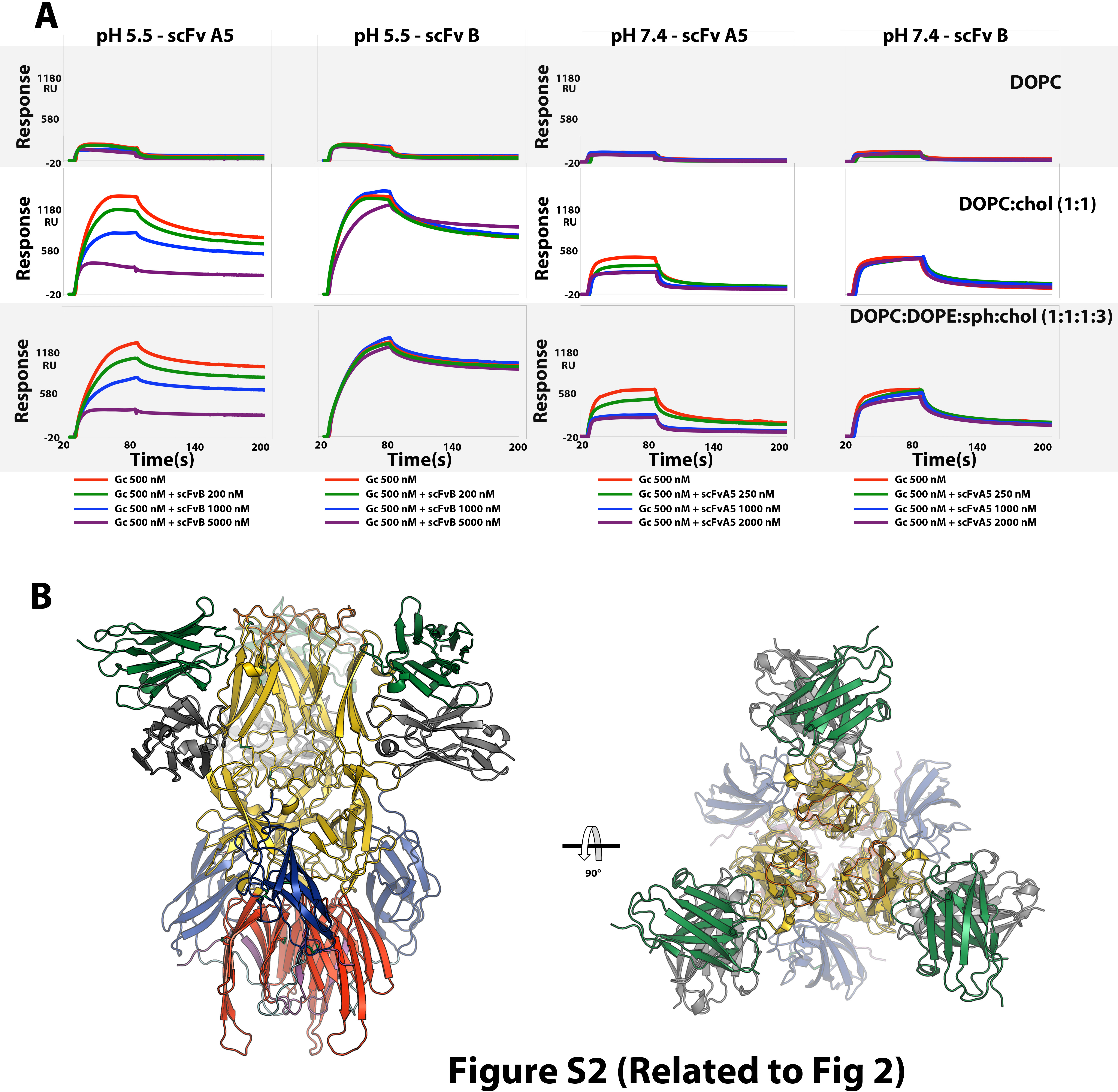

Supplement: S2 Fig — A) The rows correspond to liposomes of different compositions, as indicated in the right, which were immobilized on an L1 chip. 500 nM of Gc alone (red line) or in combination with increasing amounts (green, blue, and cyan curves) of scFv A5 (first and third column) or a control scFv (scFv “B”, second and fourth columns) were injected at pH 5.5 (first and second columns) and 7.4 (third and fourth column), as indicated in the top line, using as running buffer PBS (pH 7.4) or buffer MN (pH 5.5, see Methods for buffer composition). B) Model of three scFvs A5 interacting with a Gc trimer, showing that the binding mode is compatible with trimer formation but not with trimer insertion into membranes. (JPG) [file ppat.1005813.s002.jpg]

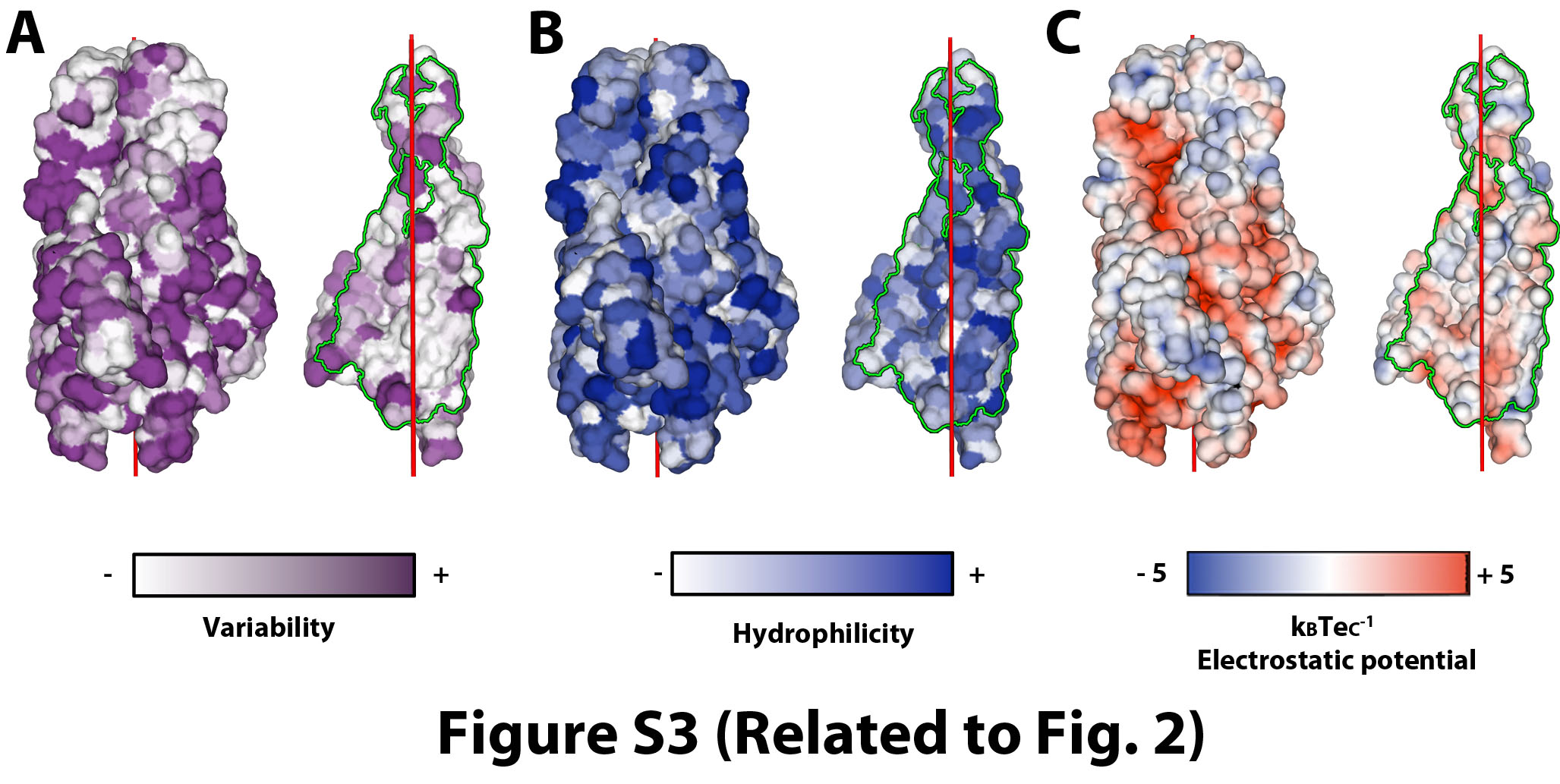

Supplement: S3 Fig — Shown are the post-fusion trimer and one of it subunits (i.e., the two foreground subunits were omitted, leaving only the one in the back, to show the buried surface within the trimer). The trimer interface is outlined in green. A) Exposed (left) and buried (right) conserved patches (white). B) Exposed (left) and buried (right) hydrophobic patches (white) and C) electrostatic surface potential. The three-fold axis is indicated in red in all the panels. (JPG) [file ppat.1005813.s003.jpg]

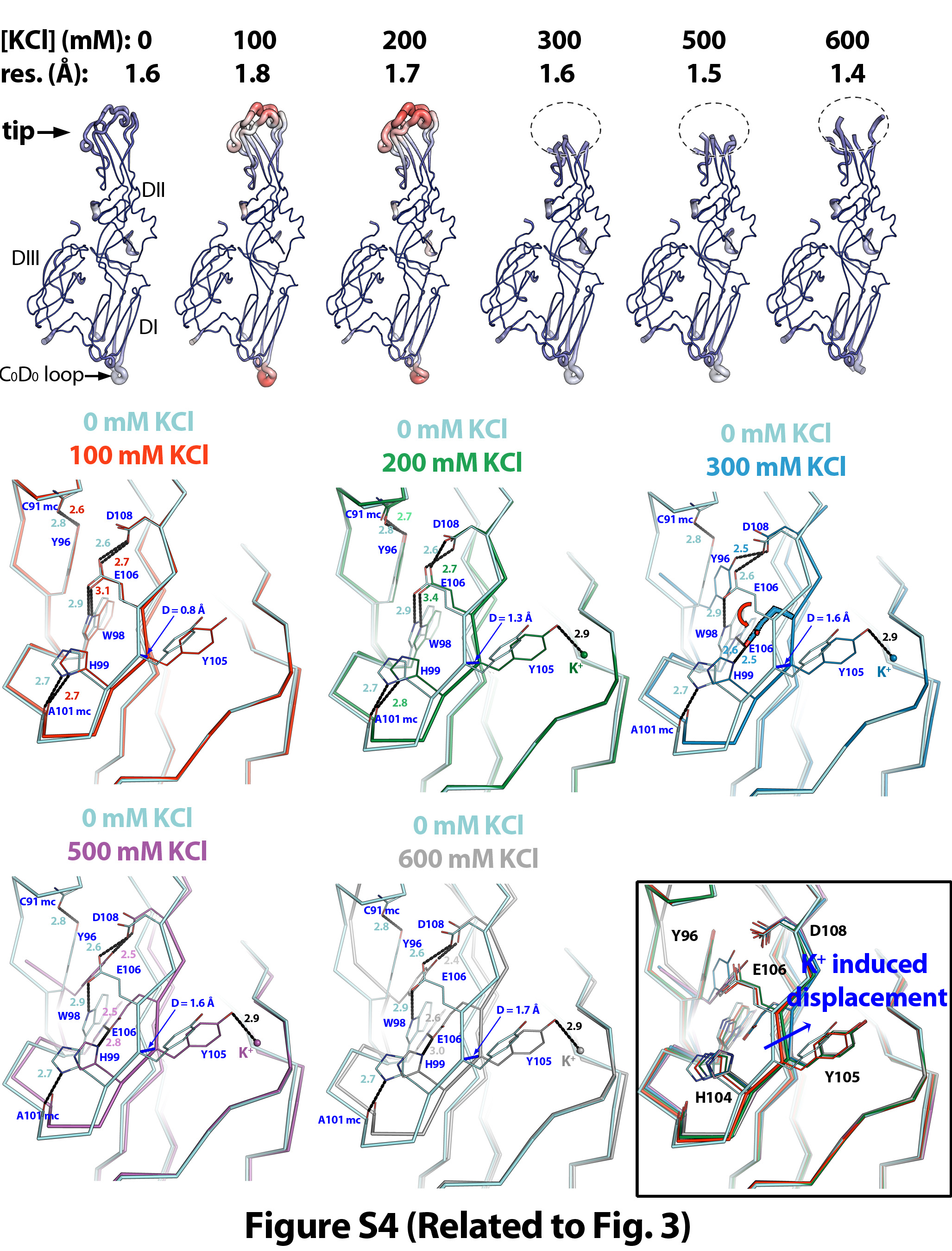

Supplement: S4 Fig — The upper panels show the structures of one Gc protomer extracted from the post-fusion trimer colored according the B-factor when crystallized at the indicated KCl concentration. We used the “cartoon putty” option in Pymol [82], in which the radius of the cartoon is proportional to the B factor, to highlight the most mobile regions. The mobility of the fusion loop increases until its electron density is lost in the structure at 300 mM KCl, with a concomitant increase in the resolution to which the crystals diffract. The middle and lower panels compare the polar network of interactions in the protein crystallized at different KCl concentrations to that in the absence of KCl. A curved red arrow in the right-hand panel, middle row, follows the movement of the Glu106 side chain, represented in thicker sticks in this panel, which is concomitant with disordering of the tip. The lower-right panel recapitulates the observed movement of Tyr105 and Glu106 by superposing all the previous panels (See S4 Movie). (JPG) [file ppat.1005813.s004.jpg]

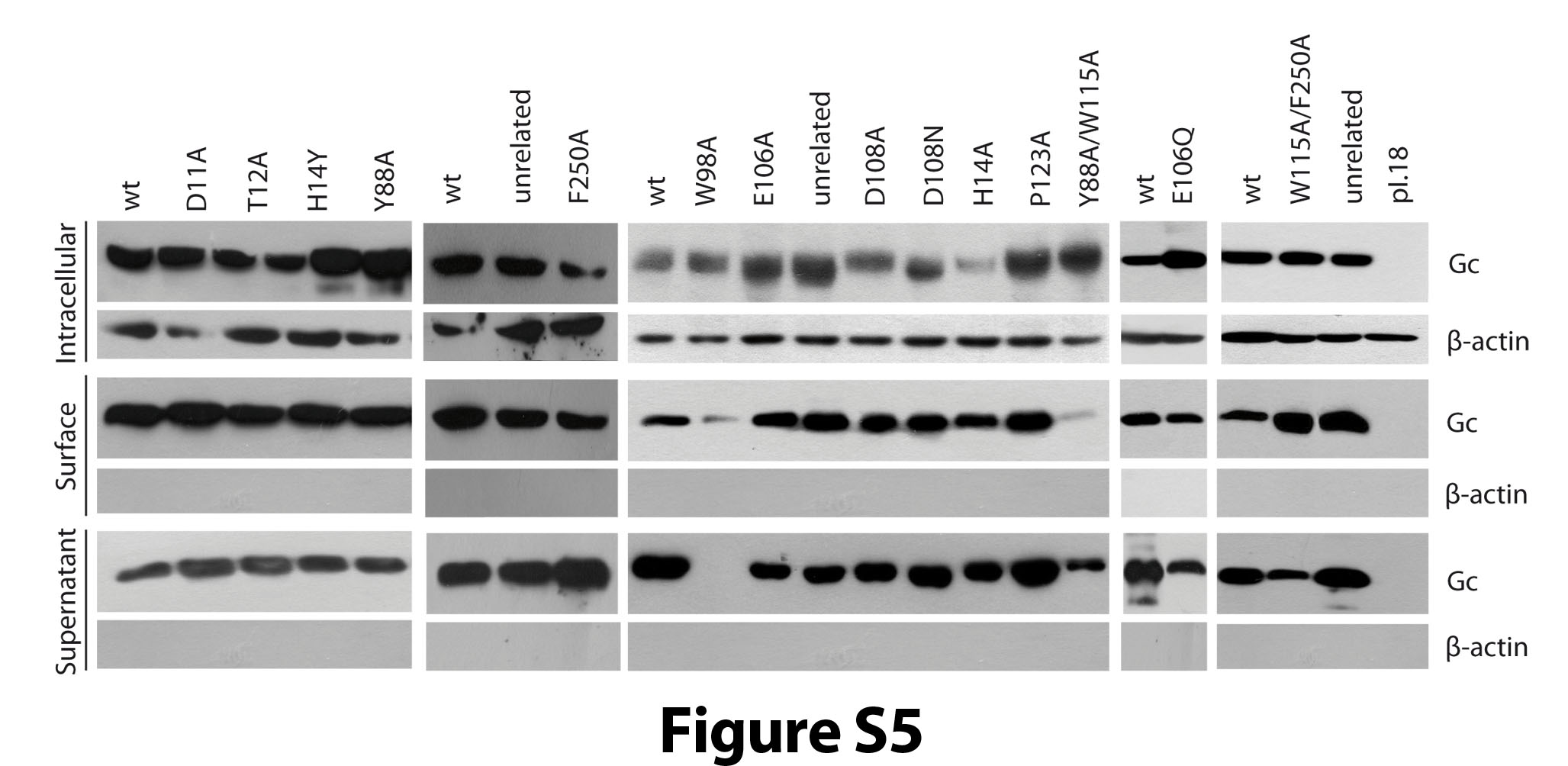

Supplement: S5 Fig — Western blot analysis using anti-Gc or anti-β-actin MAbs of different fractions obtained from 293FT cells expressing Gn and wild type or mutant Gc after surface biotinylation. The fractions correspond to the non-biotinylated fraction (intracellular proteins), the biotinylated fraction (surface proteins), or the concentrated supernatant. “Unrelated” indicates the expression of unrelated Gc mutants used as control. (JPG) [file ppat.1005813.s005.jpg]

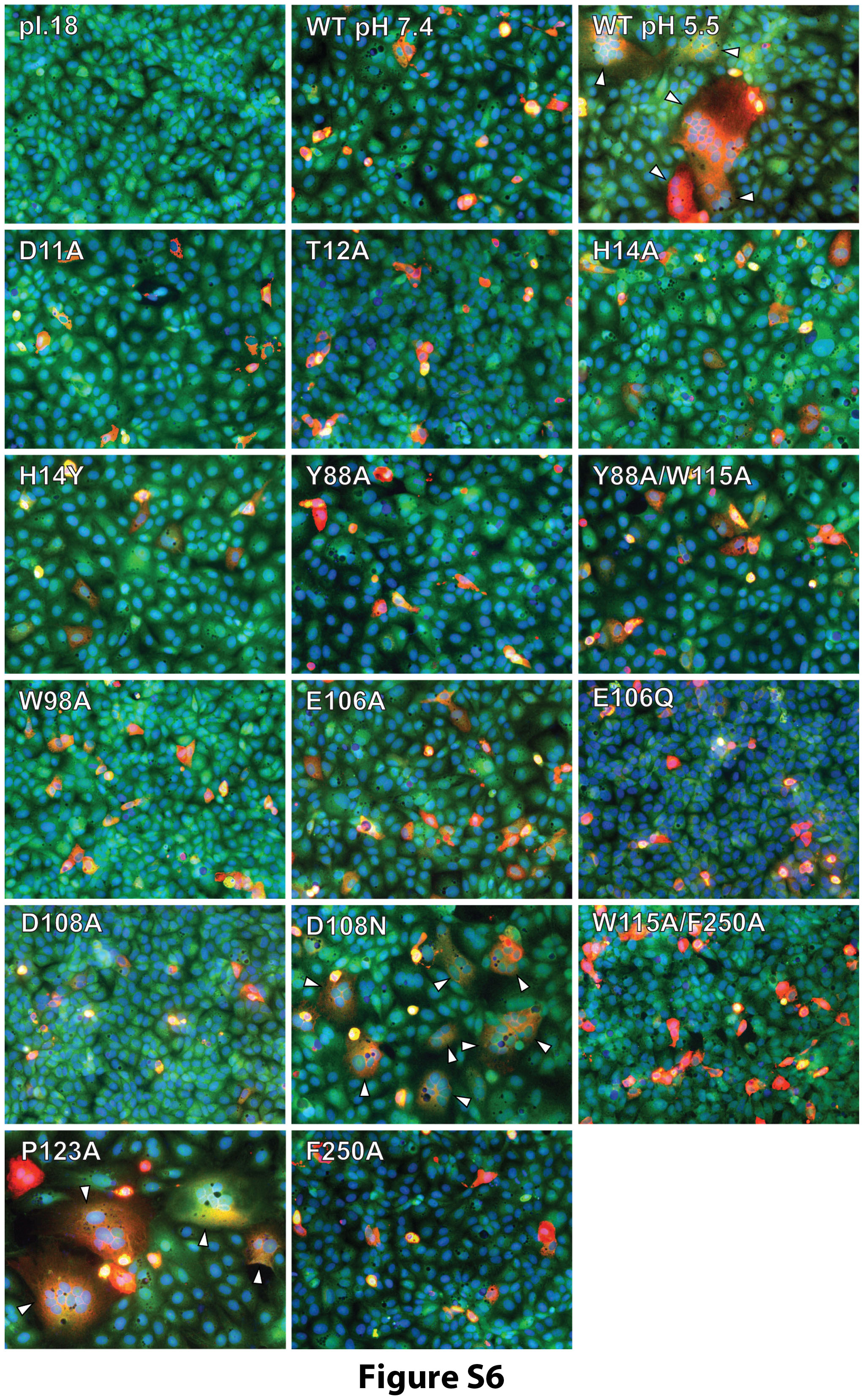

Supplement: S6 Fig — Inmunofluorescence images of Vero E6 cells expressing Gn and wild type or mutant Gc after treatment at pH 5.5. The cell cytoplasm was labelled with 5-chloromethylfluorescein diacetate (CMFDA; green fluorescence), nuclei with DAPI (blue fluorescence) and Gc was detected with anti-Gc antibody (Alexa555; red fluorescence). Cells from a partial microscopy field are shown from a representative experiment. Arrow heads indicate syncytia. (200 x magnification). (JPG) [file ppat.1005813.s006.jpg]

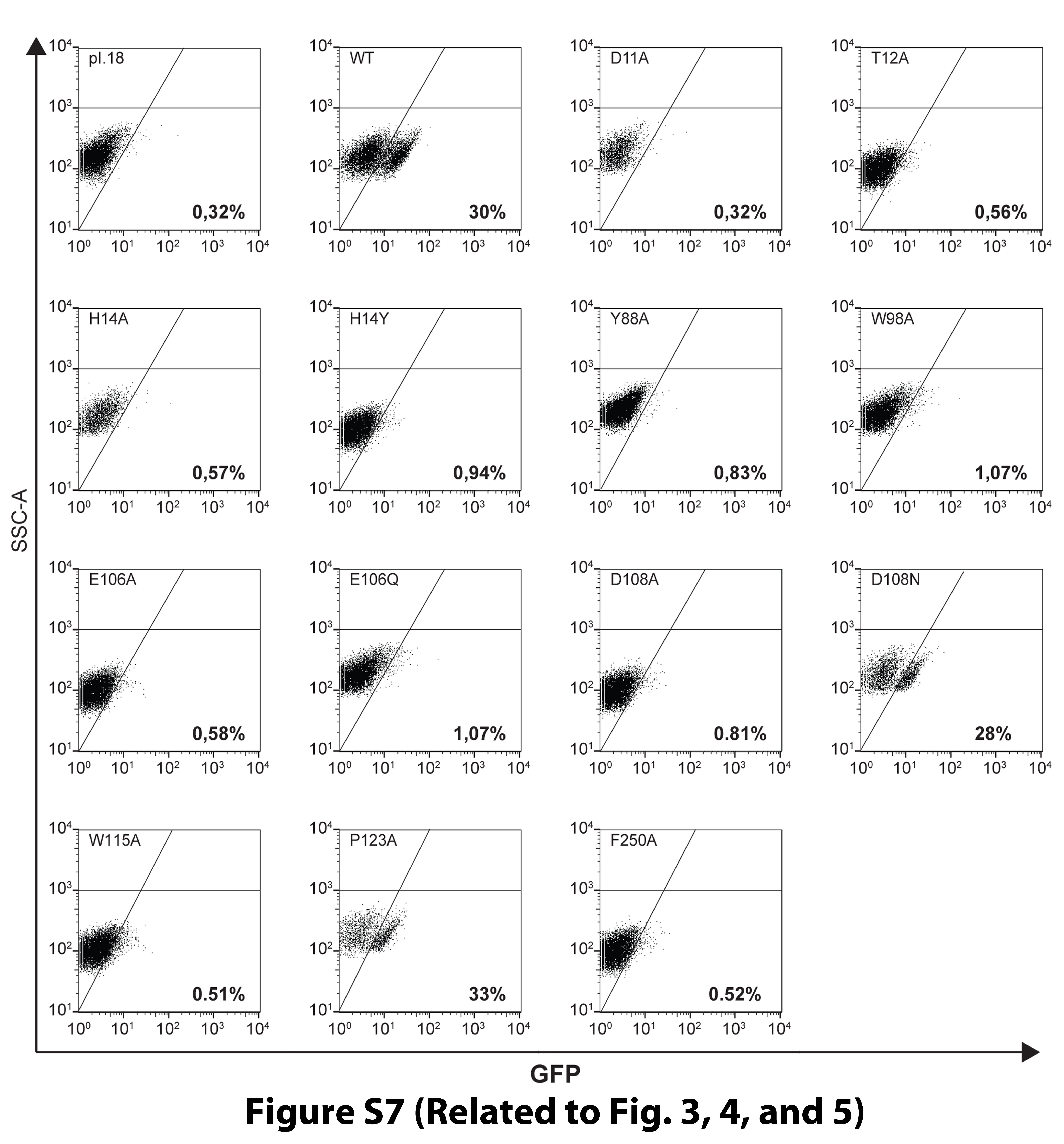

Supplement: S7 Fig — Flow cytometry gates were set for cells transduced with Mock particles (empty pI.18 vector) and the percentages shown correspond to GFP-positive cells. Data from a representative experiment are shown. (JPG) [file ppat.1005813.s007.jpg]

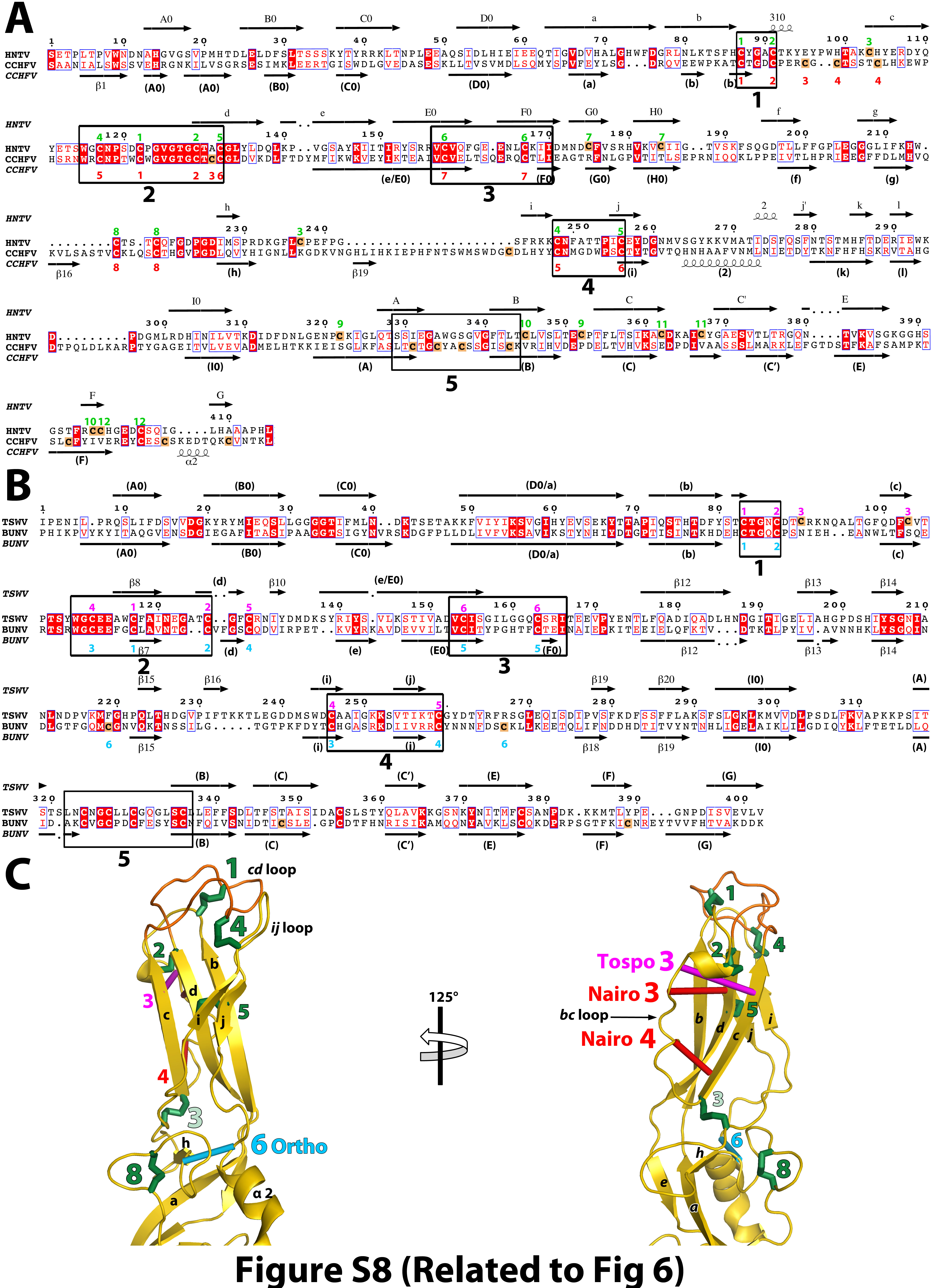

Supplement: S8 Fig — Disulfide conservation pattern across bunyavirus genera: A) Amino acid sequence alignment of Hantaan virus (HNTV, P08668.1) and Crimean-Congo Hemorrhagic Fever Virus (CCHFV, AAK52743.1). The secondary structures of HNTV are indicated above the sequences and the secondary structure predicted by PSIPRED [83, 84] for CCHFV Gc below the alignment. Shown is only the aligned region of CCHFV Gc, which is much longer. The disulfide bond connectivity in HNTV Gc is shown with green numbers and the predicted connectivity in CCHFV is shown with red numbers. The four conserved regions across hantaviruses, nairoviruses, orthobunyaviruses, and tospoviruses are boxed and numbered as in Fig 6. A 5th box indicates a region with conserved orthobunya, tospo and nairovirus cysteine residues, but less so in hantaviruses, spanning the AB loop in domain III. B) Corresponding amino acid sequence alignment of tomato spotted wilt tospovirus (TSWV, NP_049359.1) and Bunyamwera orthobunyavirus (BUNV, NP_047212.1) with the predicted secondary structures above and below the sequences and the predicted disulfide bond connectivity displayed with red and cyan numbers, respectively. C) Cartoon representation of the tip of the HNTV Gc domain II showing the location of the predicted disulfide bonds in CCHFV, BUNV, and TSWV Gc, drawn as cylinders connecting the predicted location of the corresponding cysteine residues color-coded as on the alignment of panels A and B. (JPG) [file ppat.1005813.s008.jpg]
