# Supplementary material for: Mechanistic Insight into Bunyavirus-Induced Membrane Fusion from Structure-Function Analyses of the Hantavirus Envelope Glycoprotein Gc
Source: PLoS Pathog. 2016 Oct 26;12(10):e1005813. doi: 10.1371/journal.ppat.1005813 (PMC5082683; doi:10.1371/journal.ppat.1005813)
Supplement: S1 Table — Each column represents a different diffraction data set, as indicated. The “beam line” row indicates, in parenthesis, the synchrotron to which the line belongs (ESRF: European Synchrotron radiation Facility, Grenoble, France; SOLEIL: French Synchrotron Laboratory at St Aubin, France). (DOCX) [file ppat.1005813.s009.docx]

| Table S1. Crystallographic statistics | | | | | | | | |
| --- | --- | --- | --- | --- | --- | --- | --- | --- |
|  | **Gc/scFv A5 complex** | | **Gc trimer** | | | | | |
|  |  | | **[KCl] in the crystallization condition** | | | | | |
|  | **Native** | **Sm^3+^ derivate** | **0** | **100 mM** | **200 mM** | **300 mM** | **500 mM** | **600 mM** |
| PDB code: | **5LJY** |  | **5LJZ** | **5LK0** | **5LK1** | **5LK2** | **5LK3** | **5LJX** |
| Data collection | | | | | | | | |
| Beam line | ID23-2 (ESRF) | PX1 (Soleil) | ID29 (ESRF) | ID29 (ESRF) | ID29 (ESRF) | ID29 (ESRF) | ID29 (ESRF) | ID29 (ESRF) |
| Space group | P 2_1_ 2_1_ 2 | P 2_1_ 2_1_ 2 | R 3 :H | R 3 :H | R 3 :H | R 3 :H | R 3 :H | R 3 :H |
| Unit cell parameters | | | | | | | | |
| *a* (Å) | 116.60 | 116.50 | 107.6 | 107.5 | 107.3 | 107.3 | 107.1 | 107.05 |
| *b* (Å) | 148.80 | 149.00 | 107.6 | 107.5 | 107.3 | 107.3 | 107.1 | 107.05 |
| *c* (Å) | 38.00 | 38.47 | 127.7 | 127.9 | 127.5 | 128.1 | 127.6 | 127.51 |
| Resolution (Å) | 29.76-3.00 | 38.83-3.65 | 37.64-1.60 | 37.64-1.80 | 37.55-1.70 | 37.61-1.60 | 37.52-1.50 | 37.49-1.40 |
| Last resolution bin (Å) | 3.18-3.00 | 4.00-3.65 | 1.63-1.60 | 1.84-1.80 | 1.73-1.70 | 1.63-1.60 | 1.53-1.50 | 1.42-1.40 |
| Total observations | 104053 (16955) | 292796 (50193) | 311644 (13763) | 222216 (12209) | 129396 (6349) | 313850 (14856) | 366079 (16846) | 451752 (22832) |
| Unique reflections | 13990 (2226) | 8017 (1858) | 72609 (3450) | 50655 (2948) | 56962 (2912) | 72553 (3465) | 87160 (4325) | 106974 (5328) |
| Completeness (%) | 99.9 (100.0) | 100.0 (100.0) | 99.6 (96.4) | 98.9 (96.2) | 94.5 (91.1) | 99.7 (96.8) | 99.6 (99.4) | 99.6 (100.0) |
| Redundancy | 7.4 (7.6) | 36.5 (27.0) | 4.3 (4.0) | 4.4 (4.1) | 2.3 (2.2) | 4.3 (4.3) | 4.2 (3.9) | 4.2 (4.3) |
| <I/s> | 14.0 (2.2) | 22.8 (7.3) | 13.4 (3.2) | 11.1 (3.2) | 9.3 (3.0) | 12.1 (3.5) | 8.0 (2.0) | 7.6 (1.8) |
| R_sym_ (%) | 12.1 (92.2) | 17.7 (58.1) | 5.5 (32.8) | 7.5 (36.4) | 6.1 (21.7) | 6.8 (34.3) | 9.5 (53.3) | 9.6 (60.8) |
| CC_1/2_ | 99.8 (88.6) | 99.9 (97.8) | 0.998 (0.862) | 99.6 (85.7) | 99.4 (84.0) | 99.5 (84.0) | 98.9 (64.7) | 99.0 (53.4) |
| Anomalous completeness (%) |  | 100.0 (100.0) |  |  |  |  |  |  |
| Anomalous redundancy |  | 20.0 (14.3) |  |  |  |  |  |  |
| DelAnom correlation between half-sets |  | 0.759 (0.013) |  |  |  |  |  |  |
| Refinement | | | | | | | | |
| PDB accession code |  |  |  |  |  |  |  |  |
| Resolution (Å) | 29.26-3.00 |  | 37.65-1.60 | 37.64-1.80 | 37.55-1.70 | 37.61-1.60 | 37.53-1.50 | 37.49-1.40 |
| Last resolution bin (Å) | 3.16-3.00 |  | 1.62-1.60 | 1.83-1.80 | 1.73-1.70 | 1.62-1.60 | 1.52-1.50 | 1.42-1.40 |
| Number of reflections | 13932 (1810) |  | 72584 | 50654 (2552) | 56962 (2616) | 72552 (2623) | 87148 (2763) | 106967 (3445) |
| Number of reflections Rfree | 993 (158) |  | 3690 | 2575 (159) | 2800 (109) | 3562 (133) | 4389 (130) | 5251 (166) |
| B refinement | ISOTROPIC-TLS |  | ANISOTROPIC | ISOTROPIC-TLS | ISOTROPIC-TLS | ANISOTROPIC | ANISOTROPIC | ANISOTROPIC |
| Rfactor (%) | 21.25 (31.45) |  | 13.81 (19.04) | 16.53 (20.76) | 17.72 (20.17) | 17.55 (17.52) | 17.11 (21.9) | 18.48 (23.17) |
| Rfree (%) | 26.98 (34.76) |  | 17.75 (25.95) | 19.57 (25.62) | 20.32 (25.25) | 20.81 (22.37) | 20.1 (30.0) | 20.76 (27.76) |
| Number of atoms (Mean B value in Å^2^) | | | | | | | | |
| Gc | 2920 (84.4) |  | 3268 (25.6) | 3218 (33.1) | 3247 (32.5) | 2951 (25.1) | 2829 (25.3) | 3031 (24.1) |
| scFv (H/L) | 865(90.4)/798(73.8) |  | - | - | - | - | - | - |
| MES | - |  | 12 (61.6) | - | - | - | - | - |
| Waters | 2 (47.0) |  | 445 (35.1) | 406 (35.1) | 315 (31.3) | 342 (32.7) | 368 (33.1) | 364 (31.7) |
| Hexamine-cobalt | 7 (104.6) |  | - | - | - | - | - | - |
| Na+ | - |  | 1 (18.3) | 1 (17.4) | 1 (16.3) | 1 (19.5) | 1 (15.9) | 1 (13.9) |
| K+ | - |  | - | - | 1 (23.8) | 1 (22.6) | 1 (19.2) | 1 (28.3) |
| Root mean square deviations | | | | | | | | |
| Bond lengths (Å) | 0.005 |  | 0.012 | 0.009 | 0.011 | 0.006 | 0.013 | 0.008 |
| Bond angles (º) | 1.103 |  | 1.400 | 1.153 | 1.287 | 1.047 | 1.391 | 1.279 |
| Ramachandran favored/outliers (%) | 94.29/0.17 |  | 96.45/0.24 | 96.61/0.24 | 96.67/0.48 | 96.24/0.27 | 96.29/0.53 | 95.80 / 0.79 |
